# Long noncoding RNAs are potentially involved in the degeneration of virulence in an aphid-obligate pathogen, *Conidiobolus obscurus* (Entomophthoromycotina)

**DOI:** 10.1080/21505594.2021.1938806

**Published:** 2021-06-24

**Authors:** Guofang Ye, Lvhao Zhang, Xiang Zhou

**Affiliations:** Forest Protection Department, State Key Laboratory of Subtropical Silviculture, National Joint Local Engineering Laboratory of Biopesticide High-efficient Preparation, School of Forestry and Biotechnology, Zhejiang A&F University, Hangzhou, People’s Republic of China

**Keywords:** Mycopathogen, entomophthorales, fungal virulence, fungal aging, long noncoding RNA

## Abstract

Virulence attenuation frequently occurs in *in vitro* culturing of pathogenic microbes. In this study, we investigated the total putative long noncoding RNAs (lncRNAs) in an aphid-obligate pathogen, *Conidiobolus obscurus*, and screened the differentially expressed (DE) lncRNAs and protein-coding genes involved in the virulence decline. The virulence was significantly attenuated after eight subculturing events, in which the median lethal concentration of the conidia ejected from mycelial mats relative to the bamboo aphid, *Takecallis taiwanus*, increased from 36.1 to 126.1 conidia mm^–2^, four days after inoculation. In total, 1,252 lncRNAs were identified based on the genome-wide transcriptional analysis. By characterizing their molecular structures and expression patterns, we found that the lncRNAs possessed shorter transcripts, lower expression, and fewer exons than did protein-coding genes in *C. obscurus*. A total of 410 DE genes of 329 protein-coding genes and 81 lncRNAs were identified. The functional enrichment analysis showed the DE genes were enriched in peptidase activity, protein folding, autophagy, and metabolism. Moreover, target prediction analysis of the 81 lncRNAs revealed 3,111 *cis*-regulated and 23 *trans*-regulated mRNAs, while 121 DE lncRNA-mRNA pairs were possibly involved in virulence decline. Moreover, the DE lncRNA-regulated target genes mainly encoded small heat shock proteins, secretory proteins, transporters, autophagy proteins, and other stress response-related proteins. This implies that the decline in virulence regulated by lncRNAs was likely associated with the environmental stress response of *C. obscurus*. Hence, these findings can provide insights into the lncRNA molecules of Entomophthoromycotina, with regards to virulence regulators of entomopathogens.

## Introduction

Virulence attenuation of bacterial and fungal pathogens against plants, animals, and humans is known to emerge in successive *in vitro* subcultures and long-term routine maintenance [1–4]. Changes in the chemical composition and gene expression *in vivo* have been reported to contribute to the decline in virulence [2–5]. For example, a transcriptomic rearrangement that impacted virulence was found in the continuous propagation of an intracellular bacterium, *Piscirickettsia salmonis* [3]. The decrease in protein levels in metabolism and virulence was related to the decline in successive subcultures of an entomopathogenic fungus, *Beauveria bassiana* [2]. Maintaining virulence of entomopathogens is vital when formulating applicable biocontrol agents for pest management in agroforestry systems. Hence, the regulatory mechanism of the virulence-related gene expression in entomopathogens necessitates further research.

Recently, long noncoding RNAs (lncRNAs) have been found to be related to the virulence of insect-pathogenic fungi [6]. lncRNAs are loosely defined as noncoding transcripts that are longer than 200 nucleotides and are primarily transcribed by RNA polymerase II, while lacking an open reading frame (ORF) [7]. lncRNAs are currently emerging as key regulatory components of animals, plants, and fungi. Their transcriptional regulation could act in *cis* or in *trans* modes, while either negatively or positively controlling protein-coding gene expression [7]. Multiple biological processes, including disease resistance, sexual development, stress response, and metabolite biosynthesis, were found to be related to lncRNA regulation [6, 8, 9]. Thus, lncRNAs may play a role in virulence attenuation during long-term serial culture of mycopathogens.

Most fungi in the subphylum Entomophthoromycotina (Zoopagomycota) are well-known insect- and mite-infecting pathogens [10]. They develop ballistic conidia as a means of dissemination. If the asexual conidia forcibly discharged from mycotized cadavers land on the cuticles of susceptible hosts, they germinate and secrete diverse enzymes such as glycoside hydrolases, lipases, and proteases for penetrating the host cuticle [11,12]. After exhausting the host nutritious materials, the fungi can kill the hosts and sporulate to start new infection cycle. They ultimately cause epizootics and collapse the host populations in the natural environment [13,14]. Researchers have manufactured several mycelium-based formulations, such as mycelial mats, millet granules, and alginate pellets, to apply entomophthoralean fungi for field pest control but have encountered an issue of unstable performance in control efficiency [15–17]. The aim of this study was to investigate the lncRNA molecules that are involved in the virulence decline of an aphid-obligate pathogen *Conidiobolus obscurus* (Entomophthoromycotina), using a global transcriptomic analysis. To the best of our knowledge, this is the first report to characterize the lncRNA composition in Entomophthoromycotina and to provide initial insights into its association with virulence decline.

## Materials and methods

### Successive subculturing and formulation of the mycelial mat

The isolate, *C. obscurus* ARSEF 7217, was obtained from the United States Department of Agriculture-Agricultural Research Service Collection of Entomopathogenic Fungal Cultures (Ithaca, NY, USA), which were in long-term storage at – 80°C [18]. The isolate was cultured on a rich Sabouraud dextrose agar plus yeast extract (dextrose 40, peptone 10, yeast extract 10, and agar 15 g L^–1^) for 4 d in Petri dishes at 24 ± 1°C with a 12:12 h light:dark (L:D) photoperiod. Next, the mashed culture pieces were transferred into 50 mL liquid medium of Sabouraud dextrose broth plus yeast extract and incubated for 3 d in a 150 mL flask in a shaker at 120 rpm at 24 ± 1°C. Liquid fungal cultures were successively transferred (1 mL culture was added into 50 mL fresh medium; each transfer was incubated for 3 d), following the same culture regime. The mycelia of the 1^st^, 4^th^, and 8^th^ subcultures were collected using a 0.2 µm filter and evenly poured into a 90 mm Petri dish to form a mycelial mat, while removing any excess water using sterile paper. After maintaining overnight at 24°C, the mycelial mats initiated sporulation.

### RNA extraction and transcript assembly

Three replicates were sampled from the sporulating mats that were generated from either the 1^st^ or the 8^th^ subculture, where the total RNA was extracted from all six samples using the RNAiso Plus kit (TaKaRa, Tokyo, Japan). The RNA concentration was measured using the NanoDrop2000 platform (Thermo Fisher Scientific, USA), RNA degradation and contamination (especially DNA contamination) was monitored on 1% agarose gels, and samples were sent to Biomarker Technologies Co., Ltd. (Beijing, China) for transcript sequencing. The ribosomal RNA was removed from the total RNA using a Ribo-Zero rRNA Removal Kit (Epicenter, Madison, WI, USA). A total of 1.5 µg rRNA-free RNA per sample was used to generate a sequencing library using the NEBNext® Ultra^TM^ Directional RNA Library Prep Kit from Illumina® (NEB, USA) on an Illumina Hiseq 4000 platform (BGI, Beijing, China). The raw data were deposited in CNGB Sequence Archive (CNSA) of China National GeneBank Database (CNGBdb, https://db.cngb.org/) under the accession number, CNP0001556 [19]. After trimming the adapter sequences and removing any low-quality sequences, the high-quality clean reads were aligned to the reference genome of *C. obscurus* 7217 (CNGBdb accession number: CNP0001555) using hierarchical indexing for spliced alignment of transcripts [HISAT2, 8]. Only reads with no more than two mismatches were used to generate the transcripts of each sample using StringTie software [20]. The workflow of the HISAT-StringTie analysis is shown in Fig. S1A.

### Identification of the lncRNAs

To identify the lncRNAs in *C. obscurus*, the transcripts were compared to known genes using the Cuffcompare software [21], where only the transcripts at the non-gene loci and with more than two exons were selected for further analysis. The assembled transcripts with a length of < 200 nt and those with an ORF length of >100 amino acids were excluded [9]. Moreover, the assembled transcripts with any coding potential were removed according to the evaluation of the Coding Potential Calculator (CPC), Coding Potential Assessment Tool (CPAT), and Coding-Non-Coding Index (CNCI) [22,23]. In addition, the assembled transcripts, including any known domains, were removed by comparing the sequences found in the protein family (Pfam) database [24].

### Quantification and differential expression of the lncRNAs and mRNAs

To quantify the lncRNAs and mRNAs, their expression was determined as fragments per kilobase of transcript per million mapped reads (FPKM) using StringTie 1.3.1 [20]. The differential expression analysis, of the lncRNAs and mRNAs between the subcultures, was performed using the DESeq R package 1.10.1 [25]. The resulting *P*-values were adjusted using the Benjamini and Hochberg’s approach for controlling the false discovery rate (FDR). The lncRNAs and mRNAs with an adjusted *P*-value of less than 0.01 and an absolute value of log_2_ (fold change, FC) of more than 1 were designated as differentially expressed [25].

### Predicting the target genes of lncRNAs and the functional annotation

lncRNAs located within 100 kb upstream and downstream of the corresponding mRNA were deemed to be *cis*-regulatory. lncRNA-mRNA pairs spanned beyond this range in the genomic distance, while the corresponding lncRNAs and their potential target genes were further predicted by the LncTar software [26]. If the standardized free energy was <-0.1, the lncRNA was identified as a *trans*-regulatory lncRNA.

To further examine the lncRNAs involved in the virulence changes in the subcultures, the functions of the putative lncRNA-regulated protein-coding genes were annotated using the Basic Local Alignment Search Tool (BLASTx) with an of E-value <10^–5^. The public databases of Swiss-Prot (https://www.ebi.ac.uk/uniprot), Pfam protein (http://pfam.xfam.org), Gene Ontology (GO, http://www.geneontology.org), and the Kyoto Encyclopedia of Genes and Genomes (KEGG, http://www.genome.jp/kegg/kegg2.html) platforms were used.

Functional enrichment analysis for GO terms and KEGG pathways was carried out using the R package clusterProfiler and an FDR value of ≤0.05, which indicated a significant difference [27]. Gene numbers were calculated for each GO term or pathway, and significantly enriched GO terms and pathways in DEGs compared to the genome background were defined using a hypergeometric test. The formula for calculating the *P*-value was $P=1-\overset{m-1}{\underset{i=0}{\sum }}\frac{\left(\begin{array}{c} m\\ i \end{array}\right)\left(\begin{array}{c} N-M\\ n-i \end{array}\right)}{\left(\begin{array}{c} N\\ n \end{array}\right)}$; where, *N* is the number of all genes with GO or KEGG annotation, *n* is the number of DEGs in *N, M* is the number of all genes that were annotated to certain GO terms or pathways, and m is the number of DEGs in *M*. The calculated *P*-values were subjected to FDR correction, considering FDR ≤ 0.05 as the threshold. The GO terms and pathways meeting this criterion were defined as significantly enriched GO terms or pathways in the DEGs. Protein-protein interaction (PPI) of DE protein-coding genes was predicted by blasting the genome of a related species in the STRING database (http://stringdb.org/) and then visualized in Cytoscape [28].

#### The real-time quantitative PCR (qPCR) assay

The qPCR analysis was performed to assess the transcript levels of the selected lncRNAs and mRNAs in the successive subcultures. For the analysis, the total RNA (1 µg) of each subculture was reverse-transcribed into cDNA using a PrimeScript^TM^ RT reagent kit with gDNA Eraser (TaKaRa, Japan). Next, the qPCR analysis of the cDNA samples was performed using SYBR Green PCR (SYBR Premix Ex Taq^TM^ II, TaKaRa), while the paired primers were designed and have been listed in Table S1. The PCRs were performed on a Real-Time PCR Thermal Cycler (qTOWER 2.2, Germany), while the data were analyzed using the qPCRsoft v1.1 software (Analytik Jena, Germany). Moreover, quantification of the transcript levels in the 1^st^, 4^th^, and 8^th^ subcultures was performed using at least three independent replicates. The FC was normalized relative to the expression of the internal control gene encoding elongation factor 1-alpha (*ef-1α*), which was amplified using the primers EF1-F/R (Table S1), and it was calculated using the 2^−ΔΔCt^ method [29].

### Virulence assessment

The virulence of the mycelial mats that were generated by the 1^st^, 4^th^, and 8^th^ subcultures were assessed based on multi-concentration bioassays. The test bamboo aphids (*Takecallis taiwanus*) were reared in potted bamboo plants *Chimonobambusa quadrangularis* (Fenzi) Makino, at 24 ± 1°C in a light:dark 12 h:12 h [30]. Before the bioassay, 10 adults (alatae of *T. taiwanus*) were allowed to freely produce progenies on each bamboo leaf-inclusive dish. After 24 h of reproduction, the adults were removed, leaving 17–31 nymphs of the same age on every dish for conidial inoculation. In each bioassay, the nymph cohorts on the leaf-inclusive dishes were individually exposed to a shower of primary conidia that were discharged from the sporulating mycelial mat. During the shower, a cover slip was placed near the cohort to estimate the concentration of the conidia (no. conidia mm^–2^), which were deposited onto each cohort using five microscopic counts. The duration of the exposure was controlled from several minutes to 1–2 h, where three levels of conidial concentrations were obtained in each bioassay and three replicate cohorts were showered at each concentration. Another three cohorts of nymphs that were unexposed to the conidial shower were included as blank controls in each of the aphid bioassays. All inoculated and shower-free cohorts in the leaf-inclusive dishes were maintained at 24°C and light:dark 12 h:12 h condition for 4 d. During the observation period, all cohorts were examined at 24 h intervals for mortality records and cadavers, which, whenever found, were individually mounted on glass slides to verify the mycosis of *C. obscurus* under a microscope [4].

### Phylogenic analysis

The MEGAX software suite was used to establish the phylogenetic relationships of the genes encoding the small heat shock proteins (HSP20s) [31]. The phylogenetic tree was generated using the maximum likelihood method based on the Poisson correction model, with 500 bootstrap replicates. The protein sequences and information (listed in the supplementary file) were obtained from the reference genome of *C. obscurus* 7217 (CNGBdb accession number: CNP0001555).

### Data analysis

In each aphid bioassay, the daily mortalities were corrected relative to the control mortality [4] and were then fitted to a time-concentration-mortality model as described by 32. The models were tested for goodness of fit using the Hosmer–Lemeshow test, where the fitted parameters and the associated variances of the effects of time (days post-inoculation) and the concentration, as well as the interaction between them, were used to infer the median lethal concentration (LC_50_) of *C. obscurus* conidia, ejected from the sporulating mycelial mats against the bamboo aphids. The relative expression levels of the selected DE genes in the samples of the 1^st^, 4^th^, and 8^th^ subcultures were differentiated by two-factor analysis of variance (ANOVA) with the least significant difference (LSD) test at a significance level of *P* ≤ 0.05. All analyses were conducted using an updated version of the DPS software [33].

## Results

### Decline in the virulence of the mycelial mat formulation

**Figure 1.**
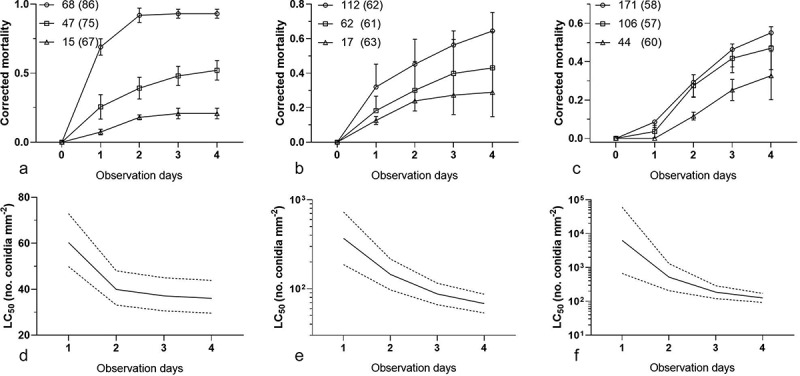
Time-concentration-mortality trends of *Takecallis taiwanus* nymphs after exposure to the shower of *Conidiobolus obscurus* conidia ejected from the mycelial mat formulation. The bamboo aphid cohorts were separately inoculated with different conidial concentrations of *C. obscurus* from the 1^st^ (a and d), 4^th^ (b and e), and 8^th^ (c and f) subcultures. (a–c) The corrected mortality at different conidial concentrations over observation days. Symbols: mean concentrations (conidia mm^–2^), to each of which three cohorts of aphids (with the total number given in parentheses) were separately exposed. Error bars reflect standard deviation. (d–f) The estimates of the median lethal concentration (LC_50_) (bold solid curve) are associated with 95% confidence limits (dash curves) over four days after conidial inoculation

No dead bamboo aphids were noted in the blank control during the 4-d observation in the bioassay. In addition, the cumulative mortalities of the inoculated aphids increased with both the conidial concentrations and the days after inoculation (Figure 1 A–C). The results showed a good fit with the time-concentration-mortality models without any significant heterogeneity based on the goodness of fit model (1^st^: Hosmer-Lemeshow *Chi2 *= 8.51, *df *= 8, *P* = 0.38; 4^th^: *Chi2 *= 1.55, *df *= 8, *P* = 0.99; 8^th^: *Chi2 *= 1.71, *df *= 9, *P* = 0.99). Based on the fitted parameters of the effects of concentration, post-shower time, and the interaction of both, the LC_50_ values of *C. obscurus* with 95% confidence intervals are presented in Figure 1 D–F. In addition, the LC_50_ of the conidia that were ejected from the mycelial mat against *T. taiwanus* increased with serial subcultures, in which the LC_50_ values increased from 36.1 to 126.1 conidia mm^–2^ four days after inoculation and eight subculturing events, indicating a decline in the virulence during the subculture process.

## *Global analysis of the lncRNAs in* C. obscurus

**Figure 2.**
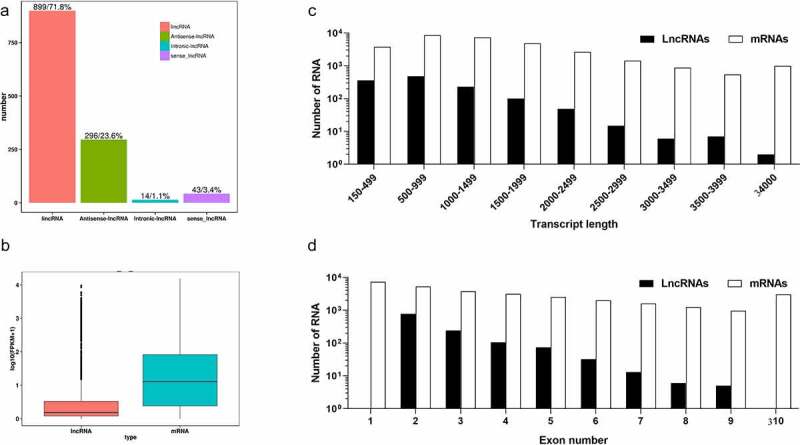
Prediction and characteristics of the long non-coding RNAs (lncRNAs) in *Conidiobolus obscurus*. (a) Number of lncRNAs across different categories: intergenic lncRNAs (lincRNAs), which are located between annotated protein-coding genes; antisense lncRNAs which are transcribed from the antisense strand; intronic lncRNAs, which overlap with the introns of annotated coding genes in either a sense or an antisense orientation; sense lncRNAs, which considered transcript variant of protein-coding mRNAs, as they overlap with a known annotated gene on the same genomic strand. (b) Expression levels of lncRNAs and the protein-coding genes in all the fungal subcultures, FPKM: fragments per kilobase of transcript per million mapped reads. (c) Transcript lengths of the protein-coding genes and lncRNAs. (d) Number of exons per transcript of the protein-coding genes and the lncRNAs

Approximately 106.2 Gb of clean reads were generated from the six libraries of *C. obscurus* subcultures, and 56.7–72.6% reads were mapped to the reference genome (Table S2). The global transcriptome analysis identified 1,252 lncRNAs based on the criteria of transcripts with >200 nt and with an ORF of <100 amino acids; analysis of CPC, CPAT, and CNCI programs; and the removal of the transcripts that encoded the conserved domains in the Pfam database (Fig. S1 B). Moreover, the identified lncRNAs were further classified as intergenic lncRNAs (lincRNAs, 899), antisense lncRNAs (296), intronic lncRNAs (14), and sense lncRNAs (43) (Figure 2 A). A total of 59,444 pairs of lncRNAs and mRNAs with either *cis*-regulation or *trans*-regulation were predicted, and each lncRNA putatively associated with an average of 47.5 molecules of mRNA (Table 1). In addition, the genome analysis of *C. obscurus* predicted 10,262 protein-coding genes, where their expression levels were notably higher than those of the lncRNAs (Figure 2 B), which was according to the FPKM values of each transcript regardless of the subcultures. Furthermore, clear differences were observed between the protein-coding genes and the lncRNAs in the distribution of the lengths of the transcripts as well as the number of exons, where approximately 79.7% of the lncRNAs had only two or three exons, while 66.1% of the lncRNAs had lengths that were less than 1 kb (Figure 2 C and D).

**Table 1.** The statistics of the putative lncRNA regulating mRNAs

	LncRNAs	LncRNA-mRNA pairs	Average pairs	Pair range
***Cis*-regulation**	1252	58,944	47	2 ~ 88
***Trans*-regulation**	346	867	2.5	1 ~ 18
**Total**	1252	59,444	47.5	1 ~ 88

One lncRNA with one of its target genes was named as one pair. Average pairs: mean number of mRNAs regulated by each lncRNA; Pair range: the minimal and maximal number of mRNAs putatively regulated by each lncRNA.

### Differential expression and functional analysis of the mRNAs and lncRNAs

**Figure 3.**
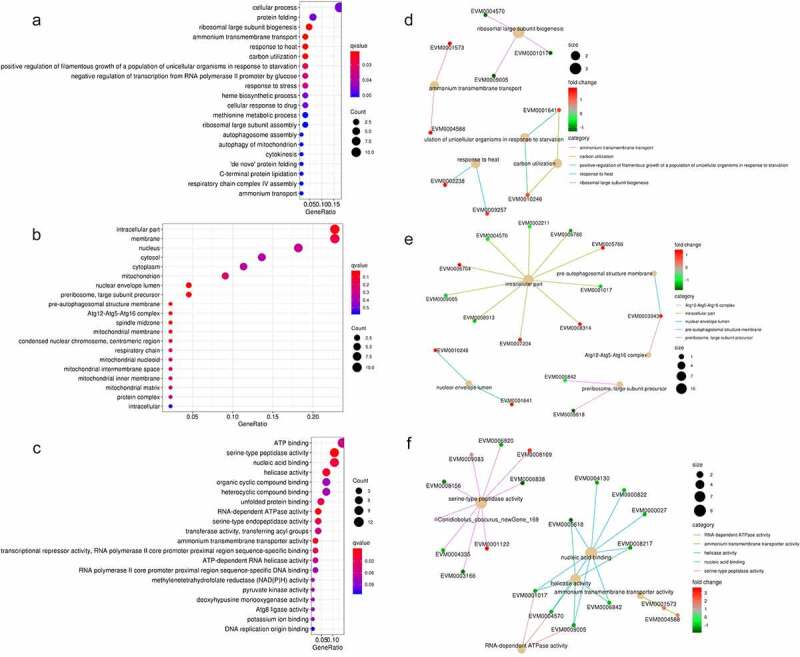
Overview of the Gene Ontology (GO) functional annotation of the differentially expressed protein-coding genes between the 1^st^ and 8^th^ subcultures. GO functional enrichment in the subcategories of biological process (a), cellular component (b), and molecular function (c). protein interactive network in the subcategories of biological process (d), cellular component (e), and molecular function (f). in A-C, the *q*-value is represented by the color of the point. the smaller the *q*-value, the closer the color is to red, and the more significant the enrichment, considering FDR≤ 0.05 as the threshold. In D-F, red: the protein-coding genes are upregulated (fold change ≥2), green: downregulated (fold change ≤-2). size of dots is related to the gene number

**Figure 4.**
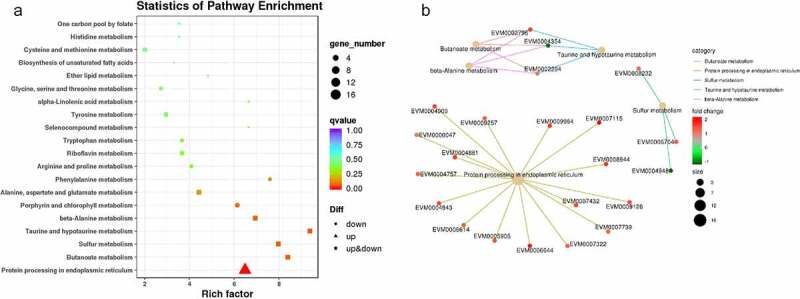
The Kyoto Encyclopedia of Genes and Genomes (KEGG) enrichment analysis of potential lncRNA-regulated protein-coding genes. KEGG functional enrichment (a): “Rich factor” refers to the ratio of the number of transcripts in the pathway entry for the differentially expressed transcripts to the total number of transcripts in the pathway entry. The larger the Rich factor, the higher the degree of enrichment. The Q-value indicates the *P*-value after multiple hypothesis test corrections ranging from 0 to 1; the closer it is to 0, the more significant the enrichment. Protein interactive network (b): red dots indicate upregulation (fold-change ≥2); green dots indicate downregulation (fold-change ≤0.5). Size of dots is related to the gene number

To determine the potential lncRNA-mRNA pairs involved in the fungal decline during subculture, a total of 410 differentially expressed (DE) genes of 329 mRNAs and 81 lncRNAs were obtained via RNA-seq between the 1^st^ and 8^th^ subcultures (Fig. S2). Of these mRNAs (164 upregulated and 165 downregulated), 258 (78.4%) were annotated based on the public databases, including 101 (30.7%) that were associated with GO terms, 122 (37.1%) that were associated with KEGG pathways, 171 (52.0%) that were annotated in Swiss-Prot, and 230 (69.9%) that were annotated in the Pfam database (Table S3). The GO terms and PPI of DE mRNAs were enriched in cellular process, protein folding, ammonium transmembrane transport, carbon utilization, response to heat, and ribosomal large subunit biogenesis in the subcategory “biological process” (Figure 3 A and D); intracellular part, membrane, Atg 12-Atg 5-Atg 16 complex, nuclear envelope lumen, pre-autophagosomal structure membrane, and preribosome in the subcategory “cellular component” (Figure 3 B and E); and ATP binding, serine-type peptidase activity, RNA-dependent ATPase activity, helicase activity, and nucleic acid binding in the subcategory “molecular function” (Figure 3 C and F). The KEGG pathway and PPI in which the DE mRNAs were involved in were enriched in protein processing in the endoplasmic reticulum, butanoate metabolism, sulfur metabolism, taurine and hypotaurine metabolism, and β-alanine metabolism (Figure 4).

Of these lncRNAs, 39 were found to be upregulated, while 42 were downregulated after successive subculturing (Table S4). A heat map was produced using hierarchical clustering analysis, in which the DE lncRNAs were distinctly self-isolated into clusters (Fig. S3). The potential targets of the DE lncRNAs were predicted to be 3,111 mRNAs that were *cis*-regulated by 81 DE lncRNAs, while 23 mRNAs were *trans*-regulated by 13 DE lncRNAs. The KEGG functional enrichment demonstrated that the DE protein-coding genes were significantly related to protein processing in the endoplasmic reticulum and SNARE interactions within the vesicular transport, as well as ubiquinone and other terpenoid-quinone biosynthesis (Fig. S4 A). The GO functional enrichment demonstrated hydrolase activity in the subcategory molecular function and intracellular part in cellular component as the most enriched terms (Fig. S4 B-D).

**Figure 5.**
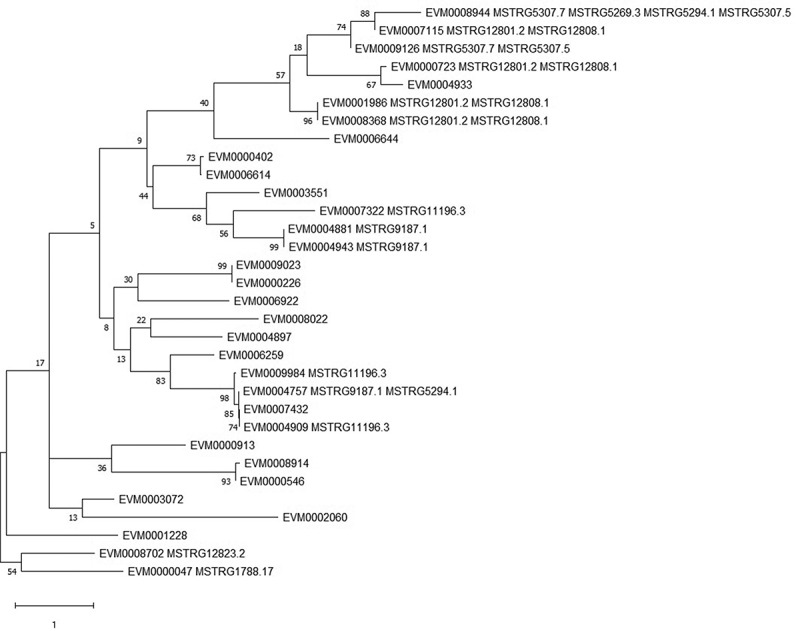
Phylogenetic tree for the 30 small heat shock proteins (HSP20s) of *Conidiobolus obscurus* based on the maximum likelihood method. The MEGAX software suite was used to infer the evolutionary histories. The tree is drawn to scale, with branch lengths measured according to the number of substitutions per site. The 12 HSP-encoding genes were putatively upregulated by the differentially expressed lncRNAs (listed behind the HSP-encoding gene ID). The rest of the HSP20s were not differentially expressed between subcultures. Another two upregulated HSP70s (EVM0008702 and EVM0000047) were used as an outgroup

**Figure 6.**
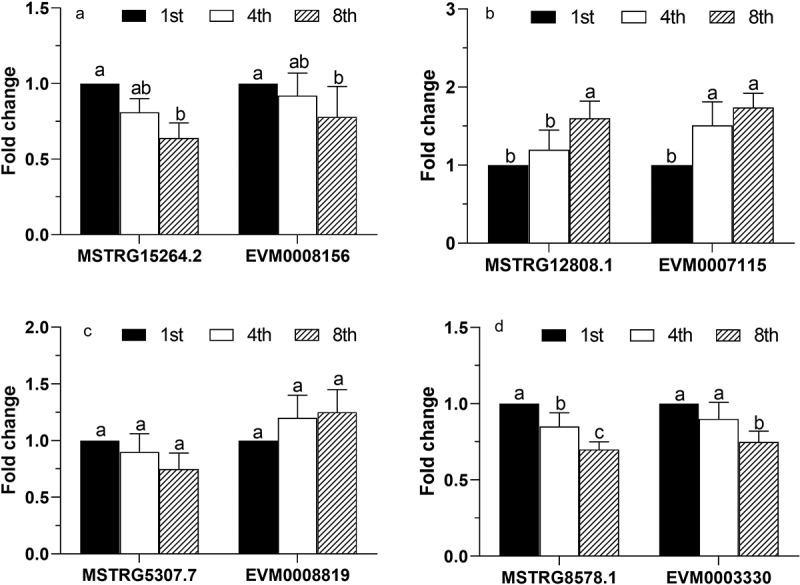
Validation of the differentially expressed mRNAs and lncRNAs by RT-qPCR. Four pairs of lncRNA-mRNAs among the 1^st^, 4^th^, and 8^th^ subcultures of *Conidiobolus obscurus* were tested. Fold changes of relative expression levels (the 1^st^ subculture as control) were calculated based on the analysis of real-time quantitative PCR. Error bars: SEM from three biological replicates. Different lowercase letters marked on the bars indicate significant differences (Fisher’s LSD, *P* < 0.05). The details of the specific primers used in this study are listed in Table S1

Furthermore, 121 putative *cis*-regulated protein-coding genes were DE across different subcultures (Table S5), most of them being small heat shock proteins (HSPs), secreted proteins, and transporters (Table 2). In particular, there were 30 genes that encoded small HSPs (HSP20/α-crystallin family) in *C. obscurus*, and all 12 HSP20-encoding DE genes were upregulated, in association with 10 DE lncRNAs (Figure 5). The four putative pairs of lncRNA-mRNA (MSTRG12808.1-EVM0007115, MSTRG15264.2-EVM0008156, MSTRG5307.7- EVM0008819, and MSTRG4282.1- EVM0003330) among the subcultures were chosen for qPCR using *ef-1α* as an internal control; the results were consistent with the RNA-seq data (Figure 6).

**Table 2.** Functional annotation of the main differentially expressed (DE) protein-coding genes that are *cis*-regulated by DE long noncoding RNAs (lncRNAs) in terms of fold change (FC)

DE protein-coding genes		Pfam annotation	DE lncRNAs	
Internal ID	Log_2_(FC)		Internal ID	Log_2_(FC)
**Heat shock protein**				
EVM0007115	2.09	Hsp20/alpha crystallin family	MSTRG.12808.1	1.81
			MSTRG.12801.2	−1.03
EVM0000723	1.81	Hsp20/alpha crystallin family	MSTRG.12808.1	1.81
			MSTRG.12801.2	−1.03
EVM0008944	1.65	Hsp20/alpha crystallin family	MSTRG.5294.1	1.56
			MSTRG.5307.5	2.53
			MSTRG.5307.7	−1.16
			MSTRG.5269.3	−2.08
EVM0004881	1.62	Hsp20/alpha crystallin family	MSTRG.9187.1	1.82
EVM0001986	1.59	Hsp20/alpha crystallin family	MSTRG.12808.1	1.81
			MSTRG.12801.2	−1.03
EVM0009984	1.59	Hsp20/alpha crystallin family	MSTRG.11196.3	−1.28
EVM0004909	1.51	Hsp20/alpha crystallin family	MSTRG.11196.3	−1.28
EVM0008368	1.48	Hsp20/alpha crystallin family	MSTRG.12808.1	1.81
			MSTRG.12801.2	−1.03
EVM0004943	1.44	Hsp20/alpha crystallin family	MSTRG.9187.1	1.82
EVM0007322	1.33	Hsp20/alpha crystallin family	MSTRG.11196.3	−1.28
EVM0004757	1.28	Hsp20/alpha crystallin family	MSTRG.9187.1	1.82
EVM0009126	1.44	Hsp20/alpha crystallin family	MSTRG.5294.1	1.56
			MSTRG.5307.5	2.53
			MSTRG.5307.7	−1.16
EVM0008702	1.14	Hsp70 protein; MreB/Mbl protein	MSTRG.12823.2	−1.91
EVM0000047	1.04	Hsp70 protein; MreB/Mbl protein	MSTRG.1788.17	1.61
**Transporter**				
EVM0005653	1.47	OPT oligopeptide transporter protein	MSTRG.8578.1	−1.49
EVM0006467	1.35	Sugar (and other) transporter; Major Facilitator Superfamily	MSTRG.9490.1	2.58
EVM0005090	1.21	IucA/IucC family; Ferric iron reductase FhuF-like transporter	MSTRG.12808.1	1.81
			MSTRG.12801.2	−1.03
EVM0008819	1.17	IucA/IucC family; Ferric iron reductase FhuF-like transporter	MSTRG.5294.1	1.56
			MSTRG.5307.5	2.53
			MSTRG.5307.7	−1.16
EVM0002578	1.18	Major Facilitator Superfamily; Sugar (and other) transporter	MSTRG.1685.1	2.22
EVM0004118	1.06	MFS/sugar transport protein	MSTRG.1685.1	2.22
EVM0005734	−1.99	Na^+^/H^+^ antiporter family	MSTRG.3953.1	−1.00
			MSTRG.3986.1	−1.87
EVM0001395	−1.47	EamA-like transporter family	MSTRG.4662.2	2.10
EVM0009886	−1.40	Ion channel regulatory protein UNC-93	MSTRG.4206.2	1.57
Secretory protein*				
EVM0005010	1.23	Glycosyl hydrolases family 18	MSTRG.4662.2	2.10
EVM0005565	1.21	Lipase (class 3)	MSTRG.8052.1	−1.69
EVM0006208	1.15	Cysteine-rich secretory protein family	MSTRG.5294.1	1.56
			MSTRG.5307.5	2.53
			MSTRG.5307.7	−1.16
			MSTRG.5269.3	−2.08
EVM0001776	−1.03	Common central domain of tyrosinase	MSTRG.12808.1	1.81
			MSTRG.12801.2	−1.03
EVM0001976	−1.01	Calcineurin-like phosphoesterase	MSTRG.5161.1	−1.22
EVM0008429	−1.16	Common central domain of tyrosinase	MSTRG.4778.2	−1.32
			MSTRG.4777.2	−1.65
EVM0008099	−1.41	Cerato-platanin	MSTRG.4206.2	1.57
EVM0003330	−1.52	Serine carboxypeptidase S28	MSTRG.8578.1	−1.49
**Metabolism**				
EVM0008232	1.23	Cys/Met metabolism PLP-dependent enzyme	MSTRG.8803.1	1.15
EVM0004178	1.34	Fatty acid desaturase	MSTRG.657.4	−1.20
			MSTRG.752.1	−1.45
			MSTRG.707.1	−1.47
EVM0002856	−1.07	Galactose oxidase, central domain	MSTRG.8052.1	−1.69
EVM0007871	−1.08	Aminotransferase class I and II	MSTRG.7571.8	3.75
EVM0007931	−1.49	Prolyl oligopeptidase family	MSTRG.4778.2	−1.32
EVM0008156	−1.51	Trypsin	MSTRG.15264.2	−1.29
EVM0003166	−1.59	Trypsin	MSTRG.10341.2	−1.80
**Others**				
EVM0007722	1.15	UDP-glucoronosyl and UDP-glucosyl transferase	MSTRG.7749.2	1.13
EVM0010159	1.13	Cytochrome P450	MSTRG.5021.2	1.89
EVM0003943	1.05	Ubiquitin-like autophagy protein Apg12; Autophagy protein Atg8	MSTRG.10639.2	2.08
EVM0008913	−1.26	Calcium/calmodulin-dependent protein kinase	MSTRG.13861.1	−1.27
EVM0007347	−2.03	Glutathione S-transferase	MSTRG.1566.1	−2.25
EVM0002155	−1.32	G protein-coupled glucose receptor regulating Gpa2	MSTRG.3953.1	−1.00
			MSTRG.3986.1	−1.87
EVM0005107	−2.69	Vacuolar import and degradation protein	MSTRG.5021.2	1.89

* Secretory proteins were screened based on their structures using the signal peptide that was predicted by SignalP v5.0 (www.cbs.dtu.dk/services/signalp) and without a membrane spanning domain. Pfam – protein family database [24]

## Discussion

The phenomenon of virulence attenuation has been widely reported in the *in vitro* culturing of entomopathogens. For example, the virulence of the rice grain formulation of *B. bassiana* decreased by 68% after 20 passages of continuous subculturing [2]. A similar result was also observed in this study where the LC_50_ values of *C. obscurus* relative to bamboo aphids increased by 249% four days after inoculation and after eight passages. Several omics studies, including genomic, transcriptomic, and proteomic studies, have been utilized to study entomopathogenicity in Entomophthoromycotina [2,34]. Several virulence factors, including metalloproteases, lipases, subtilisin-, and trypsin-like serine proteases, have been identified, most of which are secretory proteins, and the expression levels of these virulence-related genes were upregulated in the fungal cultures on insect cuticle-inclusive medium, conidia, and mycotized cadavers [11,29,35]. The decreasing expression of the virulence factors probably contributes to the decline in virulence [2,4]. In the present study, we found that the genes encoding secretory proteins of serine carboxypeptidase and tyrosinases were downregulated between the sporulating mycelial mats of subcultures, e.g., the downregulation of the serine carboxypeptidase-encoding gene (EVM0003330), putatively regulated by a DE lncRNA (MSTRG8578.1), may affect nutrient acquisition in the host hemocoel during the infection period and reduce the fungal lethal capacity [11].

In addition to the reduced levels of virulence factors that were produced, the phenotypic flexibility of the virulence can be attributed to complex regulatory networks [36]. Mitogen-activated protein kinase (MAPK) and the target of rapamycin (TOR) signaling pathways have been investigated for their roles in the expression regulation of the virulence factors of mycopathogens [2,36]. For example, Fus3-cascaded MAPK components are essential for the growth of filamentous fungi on oligotrophic substrata and the virulence of *B. bassiana* and *Metarhizium* spp [36]. Additionally, *xrn1* (encoding cytoplasmic exonuclease) the final gene of the nonsense-mediated mRNA decay (NMD) pathway, was found to determine the fate of lncRNAs in *Cordyceps militaris* and is related to the virulence attenuation [6]. Here, an lncRNA (MSTRG13861.1, Table 2)-regulated gene that putatively encodes calcium/calmodulin-dependent protein kinase was downregulated, which may operate in the calcium-triggered signaling cascade and pathogenesis [37]. Another *cis*-regulated gene putatively encoding guanine nucleotide-binding protein (G protein)-coupled glucose receptor (EVM0002155) was downregulated, which regulates Gpa2 (G protein α2) in glucose-induced cAMP signaling. Gpa2 was reported to sense extracellular carbon sources by binding to its cognate transmembrane receptor, and activate cAMP-PKA signaling (a nutrient signaling pathway) in *Saccharomyces cerevisiae* and involved in regulation of a MAPK signaling pathway in *Candida albicans* [38,39]. It may be related to the upregulated genes encoding sugar, oligopeptide, and ferric iron transporters in our study. These imply that the virulence of the entomopathogens is a comprehensive outcome in terms of their physiological status, which can be regulated by diverse pathways.

In this study, many *cis*-acting lncRNAs were found to be related to the fungal responses to environmental stresses. Remarkably, the genes belonging to the HSP20/α-crystallin family were upregulated (Table 2). HSPs were reported to be upregulated in response to metabolic and physiological stress, such as proteotoxic stress and aging [40]. Small HSPs are one of the six major families of HSPs, with molecular masses of 12–43 kDa and a highly conserved α-crystallin domain. They constitute a first line of stress defense, and have diverse roles in cellular processes, such as proteasomal degradation, autophagy, and cell differentiation [40, 41, Ungelenk et al. 42]. Stress conditions and physiological imbalances promote protein misfolding and disrupt cellular proteostasis. The upregulated small HSPs are probably involved in refolding of misfolded proteins and degradation of toxic protein aggregates and may reflect the cumulative physiological stress in subculturing. The co-existence of different forms of small HSPs (Figure 5) suggests structural specificity for endogenous proteins [43]. Moreover, the small HSPs-encoding genes putatively involved in protein processing in the endoplasmic reticulum (ko04141) may indirectly influence fungal virulence via secretion of virulence factors.

Meanwhile, an lncRNA (MSTRG10639.2)-regulated gene that putatively encodes autophagy-related proteins was also upregulated (Table 2). This result is similar with the report of successive subculturing of *B. bassiana* increasing the protein levels in autophagy and apoptosis [2]. Autophagy is linked with programmed cell death and aging, and promotes cell survival in stress conditions of growth factor deprivation and endoplasmic reticulum stress [44,45]. Thus, it implies that virulence decline could be regarded as one aspect of aging in entomopathogens.

In conclusion, this study, to the best of our knowledge, is the first to investigate the lncRNAs in entomophthoralean fungi, some of which are predicted to be involved in the virulence decline of mycelium-based formulations. These results provide a basis for investigating the roles of lncRNAs in the fungal response to continuous culturing.

## Supplementary Material

Supplemental Material
