# Supplementary material for: Long noncoding RNAs are potentially involved in the degeneration of virulence in an aphid-obligate pathogen, Conidiobolus obscurus (Entomophthoromycotina)
Source: Virulence. 2021 Jun 24;12(1):1705–16. doi: 10.1080/21505594.2021.1938806 (PMC8237998; doi:10.1080/21505594.2021.1938806)
Supplement: Supplemental Material [file KVIR_A_1938806_SM0210.zip › supplementary/supplementary fileclean.docx]

**Table S1 the designed primers for qPCR**

| Internal ID/gene | Primer Sequence | |
| --- | --- | --- |
|  | Forward | Reverse |
| LncRNAs |  |  |
| MSTRG15264.2 | TGAAATCCAAGCCTGTG | CCAGCCTAGTCCACAAC |
| MSTRG8578.1 | AGTTCTCGCAATTTCTT | TAGTTGGGCAATCTTTT |
| MSTRG12808.1 | TAAGCATAAGTTCCTCT | CATTGATCCTTTCAGTA |
| MSTRG5307.7 | TTGTTAGGCATTGAGGA | TATCTGGCTTTGGTAAA |
| mRNAs |  |  |
| EVM0007115 | CTTCAAAGTTGGAGGTCG | TAGCAGCATTAGTGAGTGG |
| EVM0003330 | GTTGGTGGTGAAAGTGA | AAATCAGGCTTAGGGTA |
| EVM0008156 | ACTCTTCGTGCTTCTCG | CGTTACGGATGTTACCC |
| EVM0008819 | GGGCTATTAGATCCTCA | TGACTTGTTCATTGGGT |
|  |  |  |
| EF1 | CGATACCTCCCTCCGCCCAG | ACAGGCGGAACGACCACAAC |

**Table S2. The numbers of mapped reads from the six samples of *Conidiobolus obscurus* subcultures**

| BMK-ID | Total Reads | Mapped Reads | Uniq Mapped Reads | Multiple Mapped Reads | Reads Map to '+' | Reads Map to '-' |
| --- | --- | --- | --- | --- | --- | --- |
| 1st1 | 113079852 | 81354935 (71.94%) | 74268163 (65.68%) | 7086772 (6.27%) | 39380304 (34.83%) | 39720150 (35.13%) |
| 1st2 | 117133180 | 85057246 (72.62%) | 77486673 (66.15%) | 7570573 (6.46%) | 41198460 (35.17%) | 41525741 (35.45%) |
| 1st3 | 121519392 | 75783626 (62.36%) | 68680587 (56.52%) | 7103039 (5.85%) | 36763399 (30.25%) | 37104864 (30.53%) |
| 8th1 | 103749878 | 66045526 (63.66%) | 60207506 (58.03%) | 5838020 (5.63%) | 32003187 (30.85%) | 32310006 (31.14%) |
| 8th2 | 120222576 | 80004783 (66.55%) | 72721319 (60.49%) | 7283464 (6.06%) | 38786667 (32.26%) | 39181199 (32.59%) |
| 8th3 | 139201422 | 78951985 (56.72%) | 72171531 (51.85%) | 6780454 (4.87%) | 38271481 (27.49%) | 38708637 (27.81%) |

**Table S3.** **The differential expression of protein-coding genes between the *Conidiobolus obscurus* subcultures** ^†^

| mRNA ID | Annotation | FPKM^‡^ | | Log_2_(FC) ^‡^ |
| --- | --- | --- | --- | --- |
|  |  | 1st | 8th |  |
| EVM0001572 | Small glutamine-rich tetratricopeptide repeat-containing protein 2 | 0 | 33.47 | 8.21 |
| EVM0008444 | Eukaryotic porin | 0 | 22.28 | 7.80 |
| EVM0008658 | C2 domain | 0 | 5.23 | 6.99 |
| EVM0007994 | kinase-like protein | 0.001 | 4.71 | 4.27 |
| EVM0001122 | putative subtilisin-like protease precursor, partial | 0.04 | 2.27 | 3.50 |
| EVM0001524 | homeobox-domain-containing protein | 5.83 | 69.36 | 3.36 |
| EVM0008109 | trypsin-like serine protease | 17.46 | 149.30 | 2.70 |
| EVM0003370 | acyl-CoA oxidase | 0.33 | 3.07 | 2.49 |
| EVM0003245 | —— | 2.44 | 19.53 | 2.19 |
| EVM0007115 | Hsp20/alpha crystallin family | 66.35 | 326.91 | 2.09 |
| EVM0000677 | putative subtilisin-like protease precursor, partial | 35.11 | 181.40 | 1.91 |
| EVM0006644 | Hsp20/alpha crystallin family | 145.61 | 740.90 | 1.83 |
| EVM0000723 | Hsp20/alpha crystallin family | 131.35 | 627.61 | 1.81 |
| EVM0001573 | ammonium transporter 2 | 26.40 | 126.13 | 1.78 |
| EVM0008805 | glycoside hydrolase family 16 protein, partial | 12.30 | 43.94 | 1.77 |
| EVM0005307 | Ring finger domain | 2.98 | 21.09 | 1.77 |
| EVM0003636 | ribonuclease T2 | 5.01 | 23.66 | 1.71 |
| EVM0008944 | Hsp20/alpha crystallin family | 43.99 | 203.67 | 1.65 |
| EVM0004588 | ammonium transporter 2 | 15.40 | 68.77 | 1.64 |
| EVM0002238 | AAA ATPase domain-containing protein | 196.94 | 793.46 | 1.63 |
| EVM0004881 | HSP20-like chaperone | 62.64 | 274.61 | 1.62 |
| EVM0001986 | Hsp20/alpha crystallin family | 413.79 | 1562.10 | 1.59 |
| EVM0009984 | HSP20-like chaperone | 55.25 | 232.68 | 1.59 |
| EVM0007316 | Arrestin (or S-antigen), N-terminal domain | 8.37 | 29.95 | 1.58 |
| EVM0003574 | ammonium transporter 2 | 14.80 | 63.33 | 1.57 |
| EVM0008271 | glycoside hydrolase family 16 protein, partial | 11.33 | 35.17 | 1.57 |
| EVM0001224 | Acetyltransferase (GNAT) domain | 5.63 | 25.92 | 1.56 |
| EVM0007213 | fatty acid alpha-hydroxylase | 129.01 | 427.16 | 1.53 |
| newGene_2221 | —— | 1.98 | 7.31 | 1.53 |
| newGene_2258 | Mpv17 / PMP22 family | 0.67 | 2.53 | 1.53 |
| EVM0002796 | CsbD-like | 99.45 | 399.95 | 1.51 |
| EVM0007432 | HSP20-like chaperone | 21.91 | 85.34 | 1.51 |
| EVM0004909 | HSP20-like chaperone | 18.73 | 72.91 | 1.51 |
| EVM0003013 | Homeobox domain | 6.60 | 22.71 | 1.50 |
| EVM0008368 | Hsp20/alpha crystallin family | 314.73 | 1153.20 | 1.48 |
| EVM0004689 | Alpha amylase, catalytic domain | 169.53 | 494.68 | 1.48 |
| EVM0005653 | OPT superfamily oligopeptide transporter | 28.50 | 109.57 | 1.47 |
| EVM0005905 | Hsp90 protein | 276.93 | 815.34 | 1.45 |
| EVM0008631 | FabD/lysophospholipase-like protein | 41.56 | 124.97 | 1.45 |
| EVM0004943 | HSP20-like chaperone | 243.35 | 965.79 | 1.44 |
| EVM0005142 | —— | 9.35 | 26.63 | 1.44 |
| EVM0009126 | Hsp20/alpha crystallin family | 149.50 | 490.58 | 1.44 |
| EVM0001457 | PH domain | 26.81 | 76.30 | 1.39 |
| EVM0001285 | glycosyltransferase family 1 protein | 0.55 | 1.96 | 1.37 |
| newGene_693 | homeobox-domain-containing protein | 0.15 | 1.67 | 1.37 |
| EVM0005927 | Activator of Hsp90 ATPase, N-terminal | 56.51 | 158.77 | 1.37 |
| EVM0007538 | acetyl-CoA synthetase-like protein | 33.27 | 118.23 | 1.36 |
| EVM0010247 | —— | 0 | 162.72 | 1.36 |
| EVM0001148 | DUF1775-domain-containing protein | 381.75 | 1049.21 | 1.35 |
| EVM0006467 | Hxt13p | 6.12 | 17.11 | 1.35 |
| EVM0006704 | Phosphoadenosine phosphosulfate reductase thioredoxin | 19.69 | 56.07 | 1.34 |
| EVM0004178 | delta 5 fatty acid desaturase | 15.53 | 40.44 | 1.34 |
| EVM0006614 | HSP20-like chaperone | 80.57 | 325.74 | 1.33 |
| EVM0007322 | HSP20-like chaperone | 136.23 | 424.99 | 1.33 |
| EVM0009257 | chaperone DnaJ | 188.34 | 504.69 | 1.32 |
| EVM0002220 | tricorn protease N-terminal domain-containing protein | 7.66 | 20.76 | 1.32 |
| EVM0005766 | tRNA intron endonuclease, catalytic C-terminal domain | 1.67 | 5.07 | 1.31 |
| EVM0001410 | Helix-loop-helix DNA-binding domain | 364.37 | 922.07 | 1.30 |
| EVM0007739 | molecular chaperone HtpG | 479.41 | 1251.37 | 1.30 |
| EVM0001641 | Zinc finger, C2H2 type | 19.77 | 51.35 | 1.28 |
| EVM0004757 | HSP20-like chaperone | 74.52 | 233.51 | 1.28 |
| EVM0008042 | DnaJ C terminal domain | 41.79 | 105.73 | 1.28 |
| EVM0007902 | 7 transmembrane receptor | 2.31 | 6.40 | 1.27 |
| EVM0001273 | —— | 1.57 | 4.01 | 1.26 |
| EVM0005577 | Di-copper centre-containing protein, partial | 7.61 | 20.53 | 1.25 |
| EVM0007382 | acyl-CoA N-acyltransferase | 1.59 | 9.27 | 1.24 |
| EVM0008302 | acyl-CoA N-acyltransferase | 4.41 | 11.25 | 1.24 |
| newGene_791 | —— | 1.85 | 7.22 | 1.24 |
| EVM0009083 | trypsin-like serine protease | 42.45 | 100.97 | 1.24 |
| EVM0008373 | Universal stress protein family | 1.45 | 4.09 | 1.23 |
| EVM0008232 | PLP-dependent transferase | 10.96 | 29.17 | 1.23 |
| EVM0005010 | carbohydrate-binding module family 19 protein | 5.55 | 14.73 | 1.23 |
| EVM0003072 | unnamed protein product | 654.38 | 1840.44 | 1.22 |
| EVM0001145 | Ctr copper transporter family | 3.71 | 11.26 | 1.22 |
| EVM0004745 | alpha/beta-hydrolase | 1.04 | 2.71 | 1.22 |
| EVM0010246 | Zinc finger, C2H2 type | 37.21 | 93.22 | 1.22 |
| EVM0002125 | —— | 194.07 | 478.36 | 1.21 |
| EVM0005744 | Histidine-specific methyltransferase, SAM-dependent | 17.10 | 42.99 | 1.21 |
| EVM0005565 | alpha/beta-hydrolase | 1.24 | 3.33 | 1.21 |
| EVM0005090 | IucA / IucC family | 9.06 | 22.39 | 1.21 |
| newGene_554 | FYVE zinc finger | 0.28 | 2.90 | 1.20 |
| EVM0001087 | —— | 6.89 | 17.34 | 1.20 |
| EVM0001703 | Threonyl and Alanyl tRNA synthetase second additional domain | 29.36 | 76.78 | 1.20 |
| EVM0003905 | HLH-domain-containing protein | 190.99 | 450.11 | 1.19 |
| EVM0002578 | Major Facilitator Superfamily | 83.81 | 197.50 | 1.18 |
| EVM0009846 | pyruvate kinase | 173.90 | 402.85 | 1.17 |
| EVM0008819 | IucA / IucC family | 13.17 | 32.15 | 1.17 |
| EVM0009485 | Protealysin propeptide | 795.48 | 1884.07 | 1.16 |
| EVM0003926 | PQ loop repeat-domain-containing protein | 3.12 | 7.65 | 1.16 |
| EVM0009354 | —— | 2.15 | 5.15 | 1.16 |
| EVM0008314 | Phytochelatin synthase | 6.26 | 14.77 | 1.16 |
| EVM0006208 | cysteine-rich secretory protein family domain-containing protein | 1.60 | 7.45 | 1.15 |
| EVM0007722 | glycosyltransferase family 1 protein | 1.27 | 3.02 | 1.15 |
| EVM0003769 | trypsin-like serine protease | 3.58 | 8.70 | 1.15 |
| EVM0008702 | heat shock protein 70 | 338.22 | 796.37 | 1.14 |
| EVM0001992 | Gelsolin repeat | 2.02 | 4.69 | 1.14 |
| EVM0010159 | cytochrome P450 | 1.22 | 2.85 | 1.13 |
| EVM0001034 | Fungal Zn(2)-Cys(6) binuclear cluster domain | 4.04 | 10.20 | 1.12 |
| EVM0003921 | Zinc finger, C2H2 type | 135.25 | 319.09 | 1.12 |
| newGene_169 | trypsin-like serine protease | 12.09 | 30.17 | 1.12 |
| EVM0008863 | family A G protein-coupled receptor-like protein | 7.11 | 16.83 | 1.11 |
| EVM0005763 | heat shock protein 60, mitochondrial precursor | 147.94 | 339.611 | 1.11 |
| EVM0001181 | DnaJ-domain-containing protein | 17.90 | 45.73 | 1.11 |
| EVM0007973 | Platelet-activating factor acetylhydrolase, isoform II | 0.77 | 3.78 | 1.11 |
| EVM0006613 | Protein of unknown function | 8.38 | 21.81 | 1.11 |
| EVM0000757 | Protein kinase domain | 175.43 | 452.17 | 1.10 |
| EVM0000997 | PTR2-domain-containing protein | 1.63 | 5.06 | 1.09 |
| EVM0004688 | AGR138Wp | 1.59 | 3.96 | 1.08 |
| newGene_1207 | Cap-specific mRNA (nucleoside-2&apos;-O-)-methyltransferase 1 | 0.45 | 1.18 | 1.08 |
| EVM0000545 | trypsin-like serine protease | 148.22 | 311.82 | 1.08 |
| EVM0005959 | ATP-dependent chaperone ClpB | 17.03 | 40.41 | 1.08 |
| EVM0007204 | Tetratricopeptide repeat | 145.47 | 309.82 | 1.08 |
| EVM0006489 | —— | 3.09 | 7.19 | 1.08 |
| EVM0004773 | Transmembrane amino acid transporter protein | 10.91 | 23.95 | 1.07 |
| EVM0006706 | AMP-binding enzyme | 1.80 | 4.03 | 1.07 |
| EVM0005202 | elongation factor 2 | 53.44 | 247.17 | 1.06 |
| EVM0004118 | major facilitator superfamily protein | 136.27 | 301.58 | 1.06 |
| EVM0002596 | family A G protein-coupled receptor-like protein | 0.68 | 2.23 | 1.05 |
| EVM0009449 | methylenetetrahydrofolate reduct | 49.62 | 107.23 | 1.05 |
| EVM0003943 | APG12-domain-containing | 6.37 | 15.46 | 1.05 |
| EVM0006161 | Di-copper centre-containing protein | 3.09 | 10.14 | 1.04 |
| EVM0000047 | 70 kDa heat shock protein 2 | 1697.87 | 3559.06 | 1.04 |
| EVM0003457 | alpha/beta-hydrolase | 25.87 | 59.84 | 1.03 |
| EVM0007180 | C6 transcription factor | 10.77 | 22.95 | 1.03 |
| EVM0002294 | CsbD-like | 11.29 | 30.84 | 1.02 |
| EVM0005885 | GTPase-activator protein for Ras-like GTPase | 0.93 | 2.07 | 1.02 |
| EVM0002897 | Hsp90 co-chaperone CDC37 | 20.39 | 42.74 | 1.02 |
| EVM0002926 | family A G protein-coupled receptor-like protein | 48.32 | 103.10 | 1.00 |
| EVM0005223 | CHAP domain | 73.89 | 156.16 | 1.00 |
| EVM0007518 | retrotransposable element | 2.40 | 1.08 | -1.00 |
| EVM0002211 | translation factor | 6.52 | 2.88 | -1.01 |
| EVM0001772 | NAD(P)-binding protein | 51.84 | 17.67 | -1.01 |
| EVM0001976 | Metallo-dependent phosphatase, partial | 4.47 | 2.05 | -1.01 |
| EVM0006608 | ParB-like nuclease domain | 70.35 | 32.20 | -1.01 |
| EVM0005563 | nli interacting factor family phosphatase | 14.24 | 6.69 | -1.02 |
| EVM0005767 | RNA recognition motif. | 57.64 | 23.99 | -1.02 |
| EVM0004354 | PLP-dependent transferase | 29.38 | 12.74 | -1.03 |
| EVM0000822 | DEAD-domain-containing protein | 8.84 | 3.55 | -1.03 |
| EVM0000556 | putative mannitol dehydrogenase | 1.68 | 0.68 | -1.03 |
| EVM0005689 | RNA recognition motif. | 54.33 | 21.87 | -1.03 |
| EVM0001776 | Di-copper centre-containing protein, partial | 2.35 | 0.88 | -1.03 |
| EVM0006329 | DEAD-domain-containing protein | 17.38 | 7.85 | -1.03 |
| EVM0008129 | WD40 repeat-like protein | 9.37 | 3.90 | -1.04 |
| EVM0002495 | —— | 11.23 | 2.26 | -1.04 |
| EVM0007139 | GATA-4/5/6 transcription factor | 5.80 | 2.24 | -1.04 |
| EVM0000833 | AUR protein kinase | 6.34 | 2.81 | -1.04 |
| EVM0003450 | P-loop containing nucleoside triphosphate hydrolase protein | 13.03 | 5.83 | -1.04 |
| EVM0006842 | DEAD/DEAH box helicase | 14.60 | 6.33 | -1.05 |
| EVM0005166 | —— | 114.68 | 51.60 | -1.05 |
| EVM0002876 | Aminotransferase class I and II | 197.76 | 88.55 | -1.05 |
| EVM0002073 | PPR repeat family | 8.08 | 3.65 | -1.06 |
| EVM0002385 | Iron-sulphur cluster biosynthesis | 85.81 | 36.28 | -1.06 |
| EVM0009247 | Transmembrane amino acid transporter protein | 32.42 | 13.78 | -1.06 |
| EVM0002985 | ankyrin, partial | 12.09 | 4.60 | -1.07 |
| EVM0005625 | —— | 11.67 | 5.10 | -1.07 |
| EVM0002856 | Galactose oxidase, central domain | 1.80 | 0.73 | -1.07 |
| EVM0007871 | Aminotransferase class I and II | 67.82 | 29.88 | -1.08 |
| EVM0007590 | Gti1/Pac2 family | 24.41 | 8.77 | -1.09 |
| EVM0004570 | DEAD-domain-containing protein | 23.28 | 8.86 | -1.09 |
| EVM0000027 | DEAD-domain-containing protein | 38.64 | 15.14 | -1.10 |
| EVM0003696 | MFS general substrate transporter | 10.77 | 4.38 | -1.10 |
| EVM0004335 | putative subtilisin-like protease precursor, partial | 4.39 | 0.90 | -1.10 |
| EVM0006268 | —— | 2.61 | 0.89 | -1.13 |
| EVM0003623 | PH-domain-containing protein | 93.23 | 39.30 | -1.13 |
| EVM0004948 | Putative Cytochrome c | 1842.82 | 667.01 | -1.14 |
| EVM0001415 | DNA/RNA polymerase | 49.70 | 20.36 | -1.14 |
| EVM0002516 | —— | 2.09 | 0.42 | -1.15 |
| EVM0007370 | Cytochrome C oxidase assembly factor 2 | 7.25 | 2.38 | -1.15 |
| EVM0008429 | Di-copper centre-containing protein | 7.09 | 2.88 | -1.16 |
| EVM0004293 | Arrestin domain-containing protein C31A2.12 | 7.00 | 2.83 | -1.16 |
| EVM0006920 | trypsin-like serine protease | 304.04 | 115.08 | -1.16 |
| EVM0004902 | MATE efflux family protein | 1.18 | 0.42 | -1.16 |
| EVM0001970 | Transmembrane amino acid transporter protein | 13.43 | 4.96 | -1.17 |
| EVM0009993 | DEAD-domain-containing protein | 25.56 | 8.57 | -1.17 |
| EVM0004130 | Zinc-finger of C2H2 type | 7.66 | 2.96 | -1.18 |
| EVM0008919 | FYVE-domain-containing protein, partial | 68.36 | 27.72 | -1.18 |
| EVM0008176 | —— | 36.44 | 14.03 | -1.18 |
| EVM0004423 | receptor/non-receptor type protein-tyrosine phosphatase, partial | 2.73 | 0.87 | -1.18 |
| EVM0009005 | DEAD-domain-containing protein | 27.17 | 9.01 | -1.19 |
| EVM0004892 | —— | 2.98 | 0.87 | -1.20 |
| EVM0001017 | DEAD-domain-containing protein | 31.63 | 11.24 | -1.21 |
| EVM0004682 | Kelch motif | 77.33 | 1.76 | -1.21 |
| EVM0004492 | Multicopper oxidase | 15.78 | 5.36 | -1.22 |
| EVM0000074 | glycoside hydrolase | 82.76 | 34.37 | -1.22 |
| EVM0008458 | Homeodomain-like DNA binding domain-containing transcription factor | 7.60 | 2.82 | -1.22 |
| EVM0003868 | amidase signature enzyme | 80.24 | 32.11 | -1.22 |
| EVM0000441 | MatE | 121.83 | 49.06 | -1.22 |
| EVM0004837 | Deoxyhypusine hydroxylase | 208.84 | 74.44 | -1.25 |
| EVM0008217 | DEAD-domain-containing protein | 37.84 | 13.31 | -1.25 |
| EVM0008913 | CAMK/CAMK1 protein kinase | 13.72 | 4.84 | -1.26 |
| EVM0002356 | HMG (high mobility group) box | 30.23 | 10.58 | -1.27 |
| EVM0008395 | DEAD-domain-containing protein | 37.62 | 11.63 | -1.28 |
| EVM0003833 | Reeler domain | 31.29 | 11.64 | -1.31 |
| EVM0007738 | lysozyme-like protein, partial | 3.66 | 0.97 | -1.31 |
| EVM0007247 | putative glucan endo-1,3-beta-glucosidase btgC | 334.38 | 120.78 | -1.32 |
| EVM0004332 | flavo protein WrbA | 2.29 | 0.68 | -1.32 |
| EVM0002155 | G protein-coupled glucose receptor regulating Gpa2 | 8.03 | 2.93 | -1.32 |
| EVM0000739 | —— | 10.21 | 3.60 | -1.32 |
| EVM0004812 | alcohol oxidase, partial | 77.10 | 24.68 | -1.33 |
| EVM0001001 | trypsin-like serine protease | 5.28 | 1.48 | -1.33 |
| EVM0009042 | Tim10/DDP family zinc finger | 55.11 | 18.93 | -1.36 |
| EVM0006957 | Helix-loop-helix DNA-binding domain | 56.68 | 19.78 | -1.37 |
| EVM0003282 | —— | 87.33 | 32.52 | -1.38 |
| EVM0009520 | Domain of unknown function | 63.21 | 22.85 | -1.38 |
| EVM0007723 | glycosyltransferase family 15 protein | 4.42 | 1.34 | -1.38 |
| EVM0005724 | Up-regulated During Septation | 30.47 | 10.95 | -1.39 |
| EVM0007704 | amidase signature enzyme | 6.15 | 2.17 | -1.40 |
| EVM0004161 | COX15/CtaA family | 213.21 | 67.04 | -1.40 |
| EVM0006780 | Possible lysine decarboxylase | 10.90 | 3.17 | -1.40 |
| EVM0009886 | MFS general substrate transporter | 7.94 | 2.08 | -1.40 |
| EVM0002361 | putative cell wall glucanase precursor, partial | 15.94 | 4.85 | -1.40 |
| EVM0006745 | Protein kinase domain | 18.26 | 5.68 | -1.41 |
| EVM0008099 | Cerato-platanin | 399.51 | 102.44 | -1.41 |
| EVM0005652 | —— | 8.10 | 1.97 | -1.45 |
| EVM0003752 | PTR2-domain-containing protein | 58.18 | 18.91 | -1.46 |
| EVM0001395 | EamA-like transporter family | 146.78 | 49.50 | -1.47 |
| EVM0009190 | —— | 2.98 | 0.69 | -1.48 |
| EVM0007931 | Prolyl oligopeptidase family | 1486.73 | 424.27 | -1.49 |
| EVM0007471 | Gti1/Pac2 family | 3.03 | 0.79 | -1.50 |
| EVM0008156 | trypsin-like protease | 42.24 | 9.63 | -1.51 |
| EVM0005272 | HSF-type DNA-binding | 17.84 | 3.40 | -1.52 |
| EVM0003330 | peptidase S28 | 90.63 | 18.72 | -1.52 |
| EVM0004796 | —— | 60.91 | 15.38 | -1.55 |
| EVM0001753 | putative triacylglycerol lipase, partial | 15.87 | 3.58 | -1.58 |
| EVM0003166 | trypsin-like protease | 32.16 | 9.21 | -1.59 |
| EVM0006227 | NDT80 / PhoG like DNA-binding family | 4.81 | 0.93 | -1.62 |
| EVM0001903 | putative triacylglycerol lipase, partial | 10.22 | 1.45 | -1.63 |
| EVM0000340 | putative subtilisin-like protease precursor, partial | 24.02 | 5.94 | -1.68 |
| EVM0001751 | putative subtilisin-like protease precursor, partial | 15.78 | 1.85 | -1.69 |
| EVM0005618 | DEAD/DEAH box helicase | 12.52 | 1.27 | -1.74 |
| EVM0008462 | Zinc-finger of C2H2 type | 19.19 | 4.54 | -1.74 |
| EVM0004721 | —— | 1.79 | 0.34 | -1.75 |
| newGene_1783 | ribosomal biogenesis protein Gar2 | 17.70 | 4.21 | -1.77 |
| EVM0006838 | trypsin-like protease | 318.55 | 74.69 | -1.79 |
| EVM0002496 | trypsin-like protease | 147.10 | 31.64 | -1.83 |
| EVM0007206 | trypsin-like serine protease | 9.49 | 1.20 | -1.83 |
| EVM0002759 | transketolase | 28.19 | 6.52 | -1.87 |
| EVM0001521 | homeobox-domain-containing protein, partial | 10.82 | 2.41 | -1.87 |
| EVM0001325 | GTP-binding protein TypA | 79.12 | 16.77 | -1.91 |
| EVM0006670 | —— | 2.54 | 0.36 | -1.92 |
| EVM0009560 | Transmembrane amino acid transporter protein | 5.13 | 0.89 | -1.94 |
| EVM0005734 | Na+/H+ antiporter family | 29.28 | 6.32 | -1.99 |
| EVM0007347 | glutathione S-transferase | 124.16 | 28.02 | -2.03 |
| EVM0005328 | DJ-1/PfpI family | 5.04 | 0.95 | -2.08 |
| EVM0000151 | Transmembrane amino acid transporter protein | 14.60 | 2.56 | -2.12 |
| EVM0006871 | glutamate--cysteine ligase catalytic subunit | 80.57 | 9.82 | -2.16 |
| EVM0003329 | Kunitz/Bovine pancreatic trypsin inhibitor domain | 10.25 | 0.59 | -2.17 |
| newGene_866 | Pet127-domain-containing protein | 3.08 | 0.36 | -2.21 |
| EVM0002535 | Na+/H+ antiporter family | 22.22 | 3.90 | -2.27 |
| EVM0007312 | Gti1/Pac2 family | 12.37 | 2.14 | -2.36 |
| EVM0000878 | Helicase conserved C-terminal domain | 12.93 | 2.19 | -2.42 |
| EVM0008556 | Transmembrane amino acid transporter protein | 2.97 | 0.36 | -2.43 |
| EVM0007389 | Kunitz/Bovine pancreatic trypsin inhibitor domain | 8.12 | 0.46 | -2.56 |
| EVM0005107 | Vacuolar import and degradation protein | 19.32 | 2.32 | -2.69 |
| EVM0004341 | putative subtilisin-like protease precursor, partial | 16.58 | 0.87 | -2.83 |
| EVM0009819 | Kunitz/Bovine pancreatic trypsin inhibitor domain | 11.50 | 0.43 | -2.85 |
| EVM0003588 | Uncharacterized protein C18B11.02c | 1.09 | 0.06 | -2.92 |
| EVM0005457 | Lytic polysaccharide mono-oxygenase, cellulose-degrading | 65.36 | 6.07 | -3.12 |

Transcript level is expressed in fragments per kilobase per million fragments (FPKM) values. FC means fold change of differentially expressed genes (DEGs) between the two libraries.

**Tables S4 The differential expression of lncRNAs between the *Conidiobolus obscurus* subcultures**

| Internal ID | FPKM | | | | | | *P* | Log2(FC) |
| --- | --- | --- | --- | --- | --- | --- | --- | --- |
|  | 1^st^-1 | 1^st^-2 | 1^st^-3 | 8^th^-1 | 8^th^-2 | 8^th^-3 |  |  |
| MSTRG.14478.1 | 0.000 | 0.000 | 0.000 | 0.251 | 0.189 | 0.230 | 0.000 | 3.821 |
| MSTRG.7571.8 | 0.000 | 0.000 | 0.000 | 0.233 | 0.561 | 0.195 | 0.000 | 3.755 |
| MSTRG.9490.1 | 0.000 | 0.000 | 0.000 | 0.132 | 0.501 | 0.132 | 0.003 | 2.580 |
| MSTRG.5307.5 | 0.218 | 0.016 | 0.032 | 0.395 | 1.128 | 1.351 | 0.001 | 2.526 |
| MSTRG.9990.2 | 0.000 | 0.000 | 0.000 | 0.201 | 0.008 | 0.054 | 0.003 | 2.507 |
| MSTRG.1685.1 | 0.000 | 0.000 | 0.000 | 0.106 | 0.138 | 0.238 | 0.009 | 2.225 |
| MSTRG.12262.1 | 0.169 | 0.159 | 0.100 | 0.338 | 0.929 | 0.948 | 0.000 | 2.113 |
| MSTRG.4662.2 | 0.001 | 0.005 | 0.002 | 0.005 | 0.028 | 1.683 | 0.014 | 2.100 |
| MSTRG.10639.2 | 0.001 | 0.005 | 0.041 | 0.015 | 0.264 | 0.512 | 0.012 | 2.081 |
| MSTRG.15488.2 | 0.053 | 0.001 | 0.115 | 16.092 | 0.144 | 0.008 | 0.017 | 2.024 |
| MSTRG.15592.2 | 2.045 | 0.854 | 2.583 | 9.118 | 4.292 | 14.445 | 0.001 | 1.998 |
| MSTRG.12132.2 | 0.006 | 0.065 | 0.046 | 0.126 | 0.233 | 0.548 | 0.007 | 1.965 |
| MSTRG.8670.2 | 0.000 | 0.003 | 0.013 | 0.001 | 0.575 | 0.007 | 0.027 | 1.892 |
| MSTRG.5021.2 | 0.006 | 0.000 | 0.188 | 0.333 | 1.253 | 0.314 | 0.022 | 1.887 |
| MSTRG.9187.1 | 0.287 | 0.218 | 0.204 | 0.692 | 0.832 | 1.825 | 0.004 | 1.816 |
| MSTRG.12808.1 | 1.657 | 1.072 | 0.352 | 2.056 | 0.885 | 18.341 | 0.025 | 1.814 |
| MSTRG.6759.2 | 0.001 | 0.009 | 0.020 | 0.019 | 0.089 | 0.122 | 0.018 | 1.757 |
| MSTRG.9970.1 | 0.183 | 0.000 | 0.062 | 0.285 | 0.286 | 0.730 | 0.036 | 1.657 |
| MSTRG.3307.2 | 0.079 | 0.000 | 0.122 | 0.329 | 0.237 | 0.391 | 0.029 | 1.639 |
| MSTRG.1788.17 | 0.043 | 0.056 | 0.015 | 0.044 | 0.040 | 0.565 | 0.047 | 1.612 |
| MSTRG.4206.2 | 0.282 | 0.159 | 0.372 | 1.476 | 0.770 | 0.583 | 0.006 | 1.575 |
| MSTRG.10728.2 | 0.167 | 0.133 | 0.000 | 0.324 | 0.577 | 0.396 | 0.042 | 1.561 |
| MSTRG.5294.1 | 2.412 | 1.294 | 2.620 | 3.288 | 8.371 | 9.844 | 0.004 | 1.559 |
| MSTRG.14730.7 | 0.031 | 0.021 | 0.007 | 0.016 | 0.133 | 0.163 | 0.050 | 1.492 |
| MSTRG.12655.2 | 0.554 | 0.279 | 0.301 | 1.306 | 1.428 | 0.868 | 0.006 | 1.443 |
| MSTRG.10856.2 | 0.499 | 0.400 | 0.826 | 1.544 | 1.603 | 1.702 | 0.001 | 1.392 |
| MSTRG.6124.1 | 3.505 | 3.993 | 4.896 | 8.232 | 11.923 | 13.387 | 0.000 | 1.375 |
| MSTRG.5365.1 | 0.650 | 1.055 | 1.837 | 1.877 | 2.743 | 6.268 | 0.031 | 1.332 |
| MSTRG.7229.1 | 0.353 | 0.288 | 0.349 | 0.586 | 1.116 | 0.928 | 0.001 | 1.330 |
| MSTRG.4293.2 | 0.635 | 0.965 | 0.293 | 1.776 | 1.905 | 1.514 | 0.011 | 1.315 |
| MSTRG.2909.1 | 0.635 | 0.369 | 0.729 | 1.092 | 1.606 | 1.711 | 0.002 | 1.278 |
| MSTRG.9799.1 | 0.246 | 0.328 | 0.252 | 0.363 | 0.993 | 0.948 | 0.016 | 1.276 |
| MSTRG.12627.2 | 1.135 | 0.873 | 1.250 | 2.576 | 2.951 | 2.245 | 0.001 | 1.211 |
| MSTRG.8803.1 | 1.830 | 1.959 | 3.040 | 6.710 | 4.093 | 5.044 | 0.008 | 1.146 |
| MSTRG.15223.2 | 0.547 | 0.852 | 0.832 | 1.848 | 2.118 | 1.318 | 0.013 | 1.144 |
| MSTRG.12287.3 | 0.256 | 0.339 | 0.193 | 0.798 | 0.401 | 0.671 | 0.035 | 1.139 |
| MSTRG.7749.2 | 0.643 | 0.209 | 0.340 | 0.728 | 1.294 | 0.992 | 0.040 | 1.130 |
| MSTRG.9862.2 | 0.725 | 0.563 | 0.469 | 0.817 | 0.994 | 2.370 | 0.029 | 1.122 |
| MSTRG.6289.1 | 2.896 | 3.756 | 1.835 | 3.902 | 6.641 | 8.366 | 0.026 | 1.059 |
| MSTRG.3953.1 | 1.310 | 1.180 | 1.969 | 0.460 | 0.921 | 0.703 | 0.025 | -1.001 |
| MSTRG.12801.2 | 0.460 | 0.620 | 0.791 | 0.320 | 0.250 | 0.268 | 0.031 | -1.027 |
| MSTRG.8779.2 | 2.525 | 2.130 | 3.788 | 1.145 | 1.723 | 0.956 | 0.026 | -1.032 |
| MSTRG.2680.2 | 1.144 | 0.824 | 0.915 | 0.590 | 0.420 | 0.252 | 0.025 | -1.040 |
| MSTRG.2881.2 | 0.226 | 0.245 | 0.182 | 0.131 | 0.038 | 0.086 | 0.048 | -1.113 |
| MSTRG.3519.1 | 0.479 | 0.557 | 0.672 | 0.267 | 0.139 | 0.274 | 0.025 | -1.157 |
| MSTRG.5307.7 | 0.656 | 0.394 | 0.269 | 0.225 | 0.082 | 0.189 | 0.045 | -1.157 |
| MSTRG.657.4 | 2.431 | 1.003 | 1.630 | 0.534 | 0.740 | 0.734 | 0.005 | -1.202 |
| MSTRG.5161.1 | 0.402 | 0.579 | 0.900 | 0.143 | 0.264 | 0.235 | 0.048 | -1.223 |
| MSTRG.13861.1 | 0.933 | 0.740 | 0.834 | 0.239 | 0.438 | 0.266 | 0.003 | -1.268 |
| MSTRG.11196.3 | 0.551 | 0.871 | 1.295 | 0.327 | 0.340 | 0.339 | 0.011 | -1.282 |
| MSTRG.15264.2 | 19.466 | 21.340 | 33.331 | 9.753 | 12.413 | 4.347 | 0.018 | -1.292 |
| MSTRG.4778.2 | 0.392 | 0.483 | 0.957 | 0.311 | 0.166 | 0.124 | 0.029 | -1.321 |
| MSTRG.13161.2 | 0.402 | 1.208 | 1.859 | 0.372 | 0.348 | 0.248 | 0.035 | -1.412 |
| MSTRG.1892.1 | 3.884 | 2.735 | 2.182 | 1.057 | 0.984 | 1.018 | 0.000 | -1.428 |
| MSTRG.2881.1 | 1.946 | 1.865 | 1.385 | 0.601 | 0.296 | 0.828 | 0.003 | -1.437 |
| MSTRG.752.1 | 1.824 | 1.321 | 1.493 | 0.462 | 0.561 | 0.563 | 0.000 | -1.449 |
| MSTRG.707.1 | 0.308 | 0.242 | 0.269 | 0.094 | 0.065 | 0.075 | 0.009 | -1.473 |
| MSTRG.8578.1 | 46.937 | 54.814 | 84.083 | 28.806 | 14.522 | 14.691 | 0.003 | -1.491 |
| MSTRG.6525.2 | 1.395 | 0.892 | 1.887 | 0.474 | 0.000 | 0.218 | 0.049 | -1.615 |
| MSTRG.14883.4 | 0.152 | 0.071 | 0.710 | 0.061 | 0.059 | 0.076 | 0.029 | -1.645 |
| MSTRG.4777.2 | 1.921 | 1.556 | 3.248 | 0.882 | 0.445 | 0.485 | 0.002 | -1.648 |
| MSTRG.8052.1 | 0.863 | 0.899 | 0.513 | 0.153 | 0.165 | 0.302 | 0.000 | -1.686 |
| MSTRG.13596.3 | 0.014 | 0.218 | 0.328 | 0.018 | 0.017 | 0.034 | 0.026 | -1.779 |
| MSTRG.10341.2 | 0.185 | 1.077 | 0.041 | 0.012 | 0.105 | 0.007 | 0.032 | -1.801 |
| MSTRG.12132.1 | 0.183 | 0.001 | 0.040 | 0.000 | 0.000 | 0.000 | 0.030 | -1.847 |
| MSTRG.12701.2 | 0.331 | 0.444 | 0.467 | 0.072 | 0.100 | 0.076 | 0.001 | -1.869 |
| MSTRG.3986.1 | 0.094 | 0.237 | 0.095 | 0.046 | 0.000 | 0.000 | 0.025 | -1.872 |
| MSTRG.12823.2 | 0.128 | 0.353 | 0.242 | 0.000 | 0.000 | 0.072 | 0.021 | -1.915 |
| MSTRG.6459.5 | 0.080 | 0.199 | 0.022 | 0.000 | 0.000 | 0.000 | 0.022 | -1.967 |
| MSTRG.3219.12 | 0.104 | 0.097 | 0.177 | 0.000 | 0.000 | 0.036 | 0.017 | -1.992 |
| MSTRG.11470.1 | 0.794 | 0.657 | 0.591 | 0.000 | 0.171 | 0.000 | 0.013 | -2.042 |
| MSTRG.5269.3 | 1.054 | 0.886 | 0.183 | 0.009 | 0.000 | 0.159 | 0.012 | -2.083 |
| MSTRG.4282.1 | 53.230 | 61.461 | 142.742 | 8.578 | 22.254 | 12.881 | 0.000 | -2.213 |
| MSTRG.11381.1 | 0.558 | 0.434 | 0.334 | 0.000 | 0.086 | 0.000 | 0.005 | -2.238 |
| MSTRG.1566.1 | 0.001 | 0.247 | 0.042 | 0.001 | 0.001 | 0.001 | 0.008 | -2.248 |
| MSTRG.15087.1 | 0.044 | 0.108 | 0.292 | 0.000 | 0.000 | 0.000 | 0.005 | -2.397 |
| MSTRG.9990.6 | 0.230 | 0.208 | 0.100 | 0.021 | 0.017 | 0.018 | 0.000 | -2.585 |
| MSTRG.9977.1 | 0.353 | 0.315 | 0.302 | 0.000 | 0.000 | 0.000 | 0.001 | -2.718 |
| MSTRG.11901.1 | 0.331 | 0.336 | 0.293 | 0.000 | 0.000 | 0.000 | 0.000 | -3.288 |
| MSTRG.5878.3 | 0.471 | 0.117 | 0.195 | 0.000 | 0.000 | 0.000 | 0.000 | -3.637 |
| MSTRG.13739.2 | 0.188 | 0.866 | 0.285 | 0.000 | 0.000 | 0.000 | 0.000 | -4.110 |

Transcript level is expressed in fragments per kilobase per million fragments (FPKM) values. FC means fold change of differentially expressed genes (DEGs) between the two libraries.

**Table S5. The functions of differentially expressed (DE) protein-coding genes putatively regulated by DE lncRNAs**

| **DE protein-coding genes** | | | **Pfam Annotation** | **DE LncRNAs** | |
| --- | --- | --- | --- | --- | --- |
| **Internal ID** |  | **Log_2_(FC)** |  | **Internal ID** | **Log_2_(FC)** |
| EVM0001572 |  | 8.21 | Homodimerisation domain of SGTA | MSTRG.3219.12 | -1.99 |
| EVM0007115 |  | 2.09 | Hsp20/alpha crystallin family | MSTRG.12808.1 | 1.81 |
|  |  |  |  | MSTRG.12801.2 | -1.03 |
| EVM0000723 |  | 1.81 | Hsp20/alpha crystallin family | MSTRG.12808.1 | 1.81 |
|  |  |  |  | MSTRG.12801.2 | -1.03 |
| EVM0008944 |  | 1.65 | Hsp20/alpha crystallin family | MSTRG.5294.1 | 1.56 |
|  |  |  |  | MSTRG.5307.5 | 2.53 |
|  |  |  |  | MSTRG.5307.7 | -1.16 |
|  |  |  |  | MSTRG.5269.3 | -2.08 |
| EVM0002238 |  | 1.63 | ATPase family associated with various cellular activities (AAA) | MSTRG.5294.1 | 1.56 |
|  |  |  |  | MSTRG.5307.5 | 2.53 |
|  |  |  |  | MSTRG.5307.7 | -1.16 |
|  |  |  |  | MSTRG.5269.3 | -2.08 |
| EVM0004881 |  | 1.62 | Hsp20/alpha crystallin family | MSTRG.9187.1 | 1.82 |
| EVM0001986 |  | 1.59 | Hsp20/alpha crystallin family | MSTRG.12808.1 | 1.81 |
|  |  |  |  | MSTRG.12801.2 | -1.03 |
| EVM0009984 |  | 1.59 | Hsp20/alpha crystallin family | MSTRG.11196.3 | -1.28 |
| EVM0007316 |  | 1.58 | Arrestin (or S-antigen) | MSTRG.9187.1 | 1.82 |
| EVM0004909 |  | 1.51 | Hsp20/alpha crystallin family | MSTRG.11196.3 | -1.28 |
| EVM0008368 |  | 1.48 | Hsp20/alpha crystallin family | MSTRG.12808.1 | 1.81 |
|  |  |  |  | MSTRG.12801.2 | -1.03 |
| EVM0005653 |  | 1.47 | OPT oligopeptide transporter protein | MSTRG.8578.1 | -1.49 |
| EVM0004943 |  | 1.44 | Hsp20/alpha crystallin family | MSTRG.9187.1 | 1.82 |
| EVM0005142 |  | 1.44 | -- | MSTRG.6759.2 | 1.76 |
| EVM0009126 |  | 1.44 | Hsp20/alpha crystallin family | MSTRG.5294.1 | 1.56 |
|  |  |  |  | MSTRG.5307.5 | 2.53 |
|  |  |  |  | MSTRG.5307.7 | -1.16 |
| EVM0005927 |  | 1.37 | Activator of Hsp90 ATPase, N-terminal | MSTRG.11381.1 | -2.24 |
| EVM0010247 |  | 1.36 | -- | MSTRG.657.4 | -1.20 |
|  |  |  |  | MSTRG.707.1 | -1.47 |
| EVM0001148 |  | 1.35 | Domain of unkown function (DUF1775) | MSTRG.9187.1 | 1.82 |
| EVM0006467 |  | 1.35 | Sugar (and other) transporter; Major Facilitator Superfamily | MSTRG.9490.1 | 2.58 |
| EVM0004178 |  | 1.34 | Fatty acid desaturase | MSTRG.657.4 | -1.20 |
|  |  |  |  | MSTRG.752.1 | -1.45 |
|  |  |  |  | MSTRG.707.1 | -1.47 |
| EVM0007322 |  | 1.33 | Hsp20/alpha crystallin family; Phosducin | MSTRG.11196.3 | -1.28 |
| EVM0004757 |  | 1.28 | Hsp20/alpha crystallin family | MSTRG.9187.1 | 1.82 |
| EVM0001273 |  | 1.26 | -- | MSTRG.12262.1 | 2.11 |
| EVM0008232 |  | 1.23 | Cys/Met metabolism PLP-dependent enzyme | MSTRG.8803.1 | 1.15 |
|  |  |  |  | MSTRG.8779.2 | -1.03 |
| EVM0005010 |  | 1.23 | Glycosyl hydrolases family 18 | MSTRG.4662.2 | 2.10 |
| EVM0004745 |  | 1.22 | -- | MSTRG.10639.2 | 2.08 |
| EVM0005565 |  | 1.21 | Lipase (class 3) | MSTRG.8052.1 | -1.69 |
| EVM0005090 |  | 1.21 | IucA / IucC family; Ferric iron reductase FhuF-like transporter | MSTRG.12808.1 | 1.81 |
|  |  |  |  | MSTRG.12801.2 | -1.03 |
| EVM0001703 |  | 1.20 | Threonyl and Alanyl tRNA synthetase second additional domain | MSTRG.7571.8 | 3.75 |
| EVM0003905 |  | 1.19 | Helix-loop-helix DNA-binding domain | MSTRG.11470.1 | -2.04 |
| EVM0002578 |  | 1.18 | Major Facilitator Superfamily; Sugar (and other) transporter | MSTRG.1685.1 | 2.22 |
| EVM0008819 |  | 1.17 | IucA / IucC family; Ferric iron reductase FhuF-like transporter | MSTRG.5294.1 | 1.56 |
|  |  |  |  | MSTRG.5307.5 | 2.53 |
|  |  |  |  | MSTRG.5307.7 | -1.16 |
| EVM0009485 |  | 1.16 | Protealysin propeptide | MSTRG.3953.1 | -1.00 |
|  |  |  |  | MSTRG.3986.1 | -1.87 |
| EVM0008314 |  | 1.16 | Phytochelatin synthase | MSTRG.8052.1 | -1.69 |
| EVM0006208 |  | 1.15 | Cysteine-rich secretory protein family | MSTRG.5294.1 | 1.56 |
|  |  |  |  | MSTRG.5307.5 | 2.53 |
|  |  |  |  | MSTRG.5307.7 | -1.16 |
|  |  |  |  | MSTRG.5269.3 | -2.08 |
| EVM0007722 |  | 1.15 | UDP-glucoronosyl and UDP-glucosyl transferase | MSTRG.7749.2 | 1.13 |
| EVM0008702 |  | 1.14 | Hsp70 protein; MreB/Mbl protein | MSTRG.12823.2 | -1.91 |
| EVM0010159 |  | 1.13 | Cytochrome P450 | MSTRG.5021.2 | 1.89 |
| EVM0001034 |  | 1.12 | Fungal Zn(2)-Cys(6) binuclear cluster domain | MSTRG.9862.2 | 1.12 |
| EVM0003921 |  | 1.12 | C2H2-type zinc finger | MSTRG.7229.1 | 1.33 |
| EVM0008863 |  | 1.11 | 7 transmembrane receptor (rhodopsin family) | MSTRG.14883.4 | -1.64 |
| EVM0004118 |  | 1.06 | MFS/sugar transport protein | MSTRG.1685.1 | 2.22 |
| EVM0003943 |  | 1.05 | Ubiquitin-like autophagy protein Apg12; Autophagy protein Atg8 ubiquitin like | MSTRG.10639.2 | 2.08 |
| EVM0000047 |  | 1.04 | Hsp70 protein;; MreB/Mbl protein | MSTRG.1788.17 | 1.61 |
| EVM0003457 |  | 1.03 | -- | MSTRG.10639.2 | 2.08 |
| EVM0002926 |  | 1.00 | -- | MSTRG.9799.1 | 1.28 |
| EVM0002211 |  | -1.01 | Telomere recombination; Putative GTP-binding controlling metal-binding | MSTRG.12808.1 | 1.81 |
|  |  |  |  | MSTRG.12801.2 | -1.03 |
| EVM0001772 |  | -1.01 | N-terminal domain of oxidoreductase; Zinc-binding dehydrogenase | MSTRG.752.1 | -1.45 |
| EVM0001976 |  | -1.01 | Calcineurin-like phosphoesterase | MSTRG.5161.1 | -1.22 |
| EVM0005563 |  | -1.02 | NLI interacting factor-like phosphatase | MSTRG.657.4 | -1.20 |
| EVM0005767 |  | -1.02 | RNA recognition motif | MSTRG.12655.2 | 1.44 |
| EVM0005689 |  | -1.03 | RNA recognition motif | MSTRG.6289.1 | 1.06 |
| EVM0001776 |  | -1.03 | Common central domain of tyrosinase | MSTRG.12808.1 | 1.81 |
|  |  |  |  | MSTRG.12801.2 | -1.03 |
| EVM0000833 |  | -1.04 | Protein tyrosine kinase | MSTRG.2680.2 | -1.04 |
| EVM0005166 |  | -1.05 | -- | MSTRG.1685.1 | 2.22 |
| EVM0002073 |  | -1.06 | Pentatricopeptide repeat domain | MSTRG.10856.2 | 1.39 |
| EVM0002385 |  | -1.06 | Iron-sulphur cluster biosynthesis | MSTRG.12655.2 | 1.44 |
| EVM0005625 |  | -1.07 | -- | MSTRG.3219.12 | -1.99 |
| EVM0002856 |  | -1.07 | Galactose oxidase, central domain | MSTRG.8052.1 | -1.69 |
| EVM0007871 |  | -1.08 | Aminotransferase class I and II | MSTRG.7571.8 | 3.75 |
| EVM0004570 |  | -1.09 | DEAD/DEAH box helicase | MSTRG.12132.1 | -1.85 |
|  |  |  |  | MSTRG.12132.2 | 1.97 |
| EVM0000027 |  | -1.10 | DEAD/DEAH box helicase | MSTRG.7229.1 | 1.33 |
| EVM0003623 |  | -1.13 | PH domain | MSTRG.752.1 | -1.45 |
|  |  |  |  | MSTRG.707.1 | -1.47 |
| EVM0001415 |  | -1.14 | DNA-dependent RNA polymerase | MSTRG.3519.1 | -1.16 |
| EVM0008429 |  | -1.16 | Common central domain of tyrosinase | MSTRG.4778.2 | -1.32 |
|  |  |  |  | MSTRG.4777.2 | -1.65 |
| EVM0004902 |  | -1.16 | MatE | MSTRG.6289.1 | 1.06 |
| EVM0008919 |  | -1.18 | FYVE zinc finger | MSTRG.657.4 | -1.20 |
| EVM0000441 |  | -1.22 | MatE | MSTRG.2680.2 | -1.04 |
| EVM0008913 |  | -1.26 | Protein tyrosine kinase | MSTRG.13861.1 | -1.27 |
| EVM0002155 |  | -1.32 | G protein-coupled glucose receptor regulating Gpa2 | MSTRG.3953.1 | -1.00 |
|  |  |  |  | MSTRG.3986.1 | -1.87 |
| EVM0009042 |  | -1.36 | Tim10/DDP family zinc finger | MSTRG.752.1 | -1.45 |
|  |  |  |  | MSTRG.707.1 | -1.47 |
| EVM0009886 |  | -1.40 | Ion channel regulatory protein UNC-93 | MSTRG.4206.2 | 1.57 |
| EVM0008099 |  | -1.41 | Cerato-platanin | MSTRG.4206.2 | 1.57 |
| EVM0001395 |  | -1.47 | EamA-like transporter family | MSTRG.4662.2 | 2.10 |
| EVM0007931 |  | -1.49 | Prolyl oligopeptidase family | MSTRG.4778.2 | -1.32 |
|  |  |  |  | MSTRG.4777.2 | -1.65 |
| EVM0007471 |  | -1.50 | Gti1/Pac2 family | MSTRG.9799.1 | 1.28 |
|  |  |  |  | MSTRG.9862.2 | 1.12 |
| EVM0008156 |  | -1.51 | Trypsin | MSTRG.15264.2 | -1.29 |
| EVM0003330 |  | -1.52 | Serine carboxypeptidase S28 | MSTRG.8578.1 | -1.49 |
| EVM0004796 |  | -1.55 | -- | MSTRG.5878.3 | -3.64 |
| EVM0003166 |  | -1.59 | Trypsin | MSTRG.10341.2 | -1.80 |
| EVM0008462 |  | -1.74 | Zinc-finger of C2H2 type | MSTRG.5365.1 | 1.33 |
| EVM0004721 |  | -1.75 | -- | MSTRG.10728.2 | 1.56 |
| Co_1783 |  | -1.77 | RNA recognition motif | MSTRG.5021.2 | 1.89 |
| EVM0005734 |  | -1.99 | Na+/H+ antiporter family | MSTRG.3953.1 | -1.00 |
|  |  |  |  | MSTRG.3986.1 | -1.87 |
| EVM0007347 |  | -2.03 | Glutathione S-transferase | MSTRG.1566.1 | -2.25 |
| EVM0006871 |  | -2.16 | Glutamate-cysteine ligase | MSTRG.8052.1 | -1.69 |
| EVM0000878 |  | -2.42 | Helicase conserved C-terminal domain | MSTRG.3219.12 | -1.99 |
| EVM0005107 |  | -2.69 | Vacuolar import and degradation protein | MSTRG.5021.2 | 1.89 |

FC means fold change of differentially expressed genes (DEGs) between the two libraries.


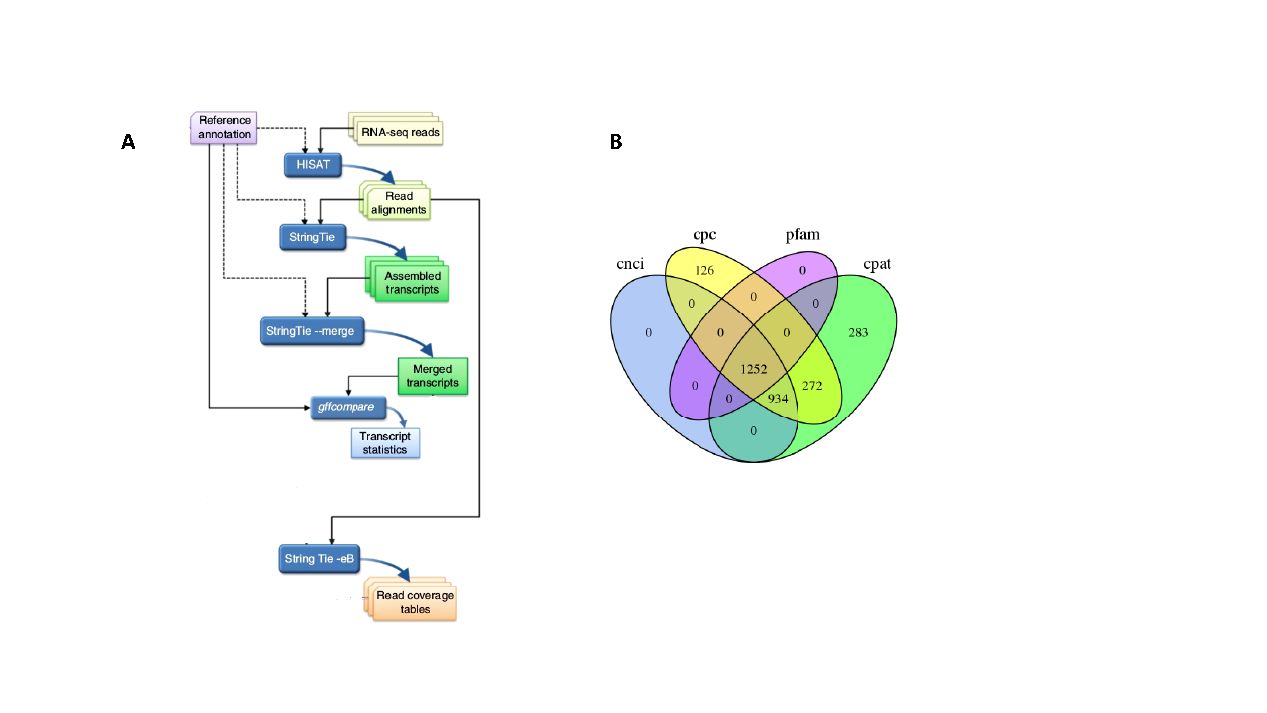


**Fig. S1.** Overview of RNA sequencing. (A) The workflow of RNA-seq. (B) Venn diagram analysis of the numbers of candidate lncRNAs filtered by CPC, CPAT, CNCI, and comparison with the Pfam database.


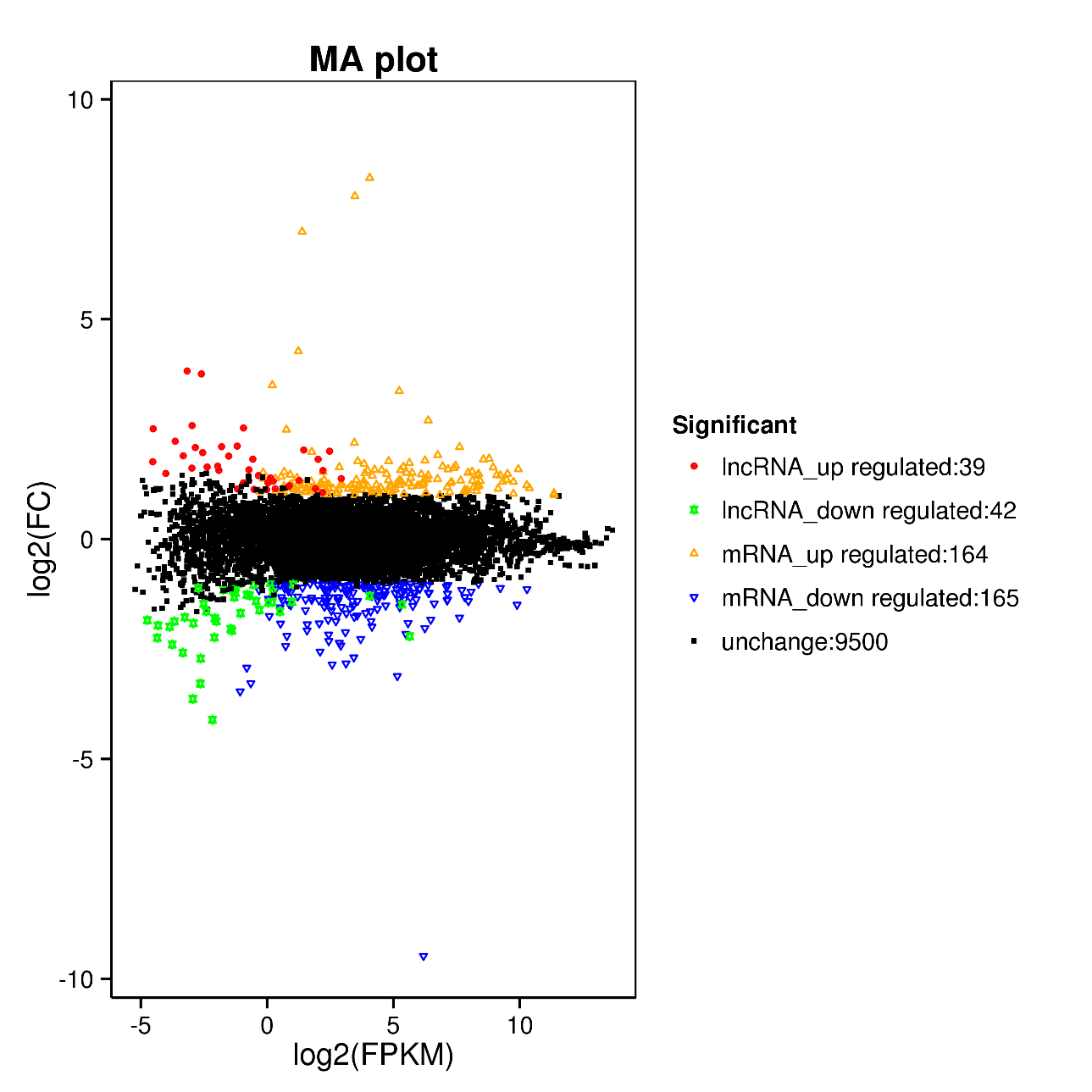


**Fig. S2** Expression levels of the long non-coding RNAs (lncRNAs) and the protein-coding genes (mRNAs) in *Conidiobolus obscurus* subcultures. The scatter plot shows the relationship between log the fragments per kilobase per million fragments (log_2_FPKM) and the log fold change (log_2_FC) for each differentially expressed gene (FC≥2), which are based on the sequencing of the RNA that was extracted from the 8^th^ subculture when compared to the 1^st^ subculture. Each symbol represents a single gene, the black circles indicate the genes without differential expression (unchange, gene number: 9500), while the colored symbols indicate genes that are differentially expressed at a false discovery rate of ≤0.05 (of these mRNAs,164 upregulated and 165 downregulated; Of these lncRNAs, 39 were found to be upregulated, while 42 were downregulated).


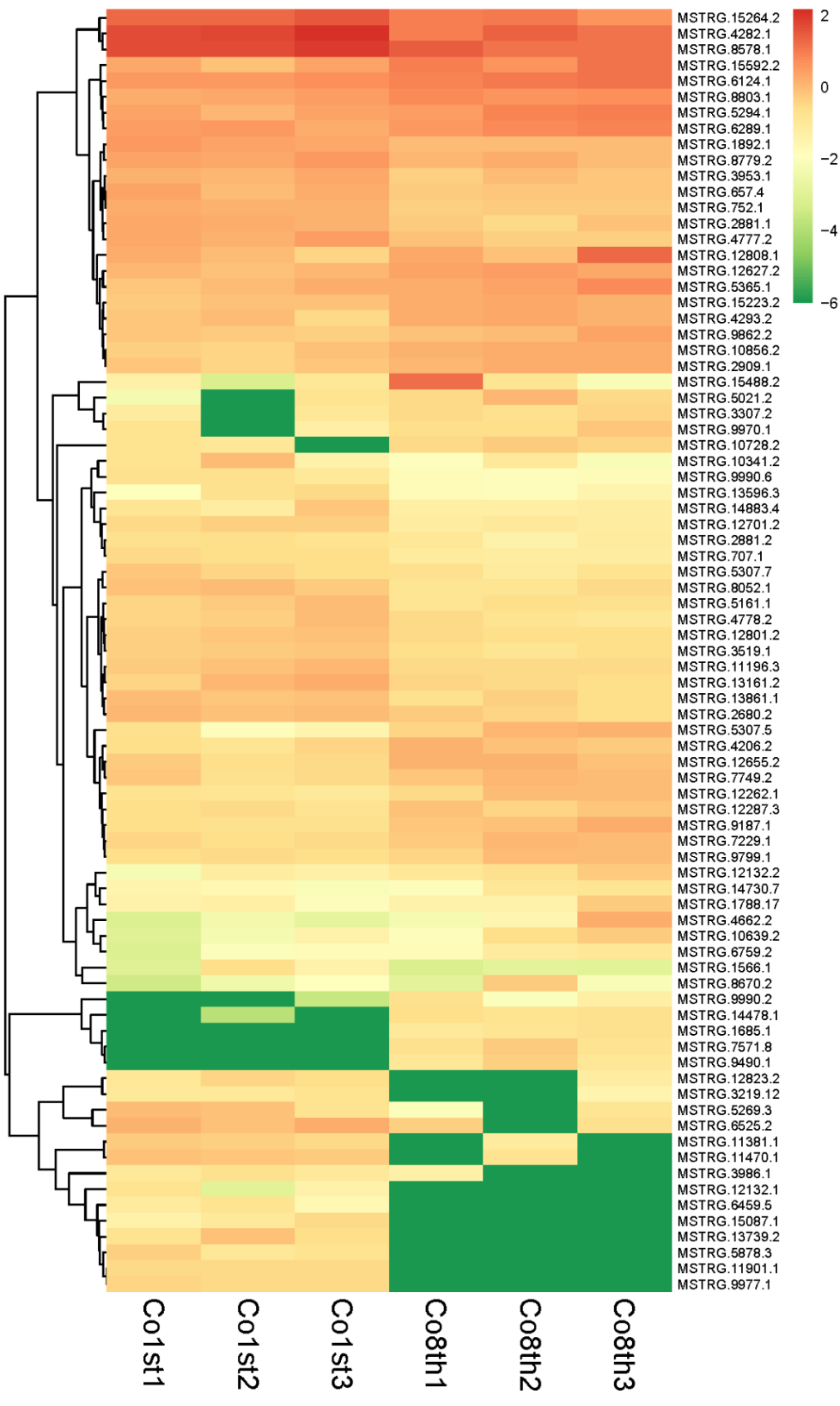


**Fig. S3** Heat map of the transcription patterns of the differentially expressed long non-coding RNAs (lncRNAs) among the six samples of the *Conidiobolus obscurus* subcultures. The patterns were obtained from hierarchical clustering and the normalization of the log_10_ of the fragments per kilobase per million (FPKM), which were used to determine the gene expression levels across different samples.


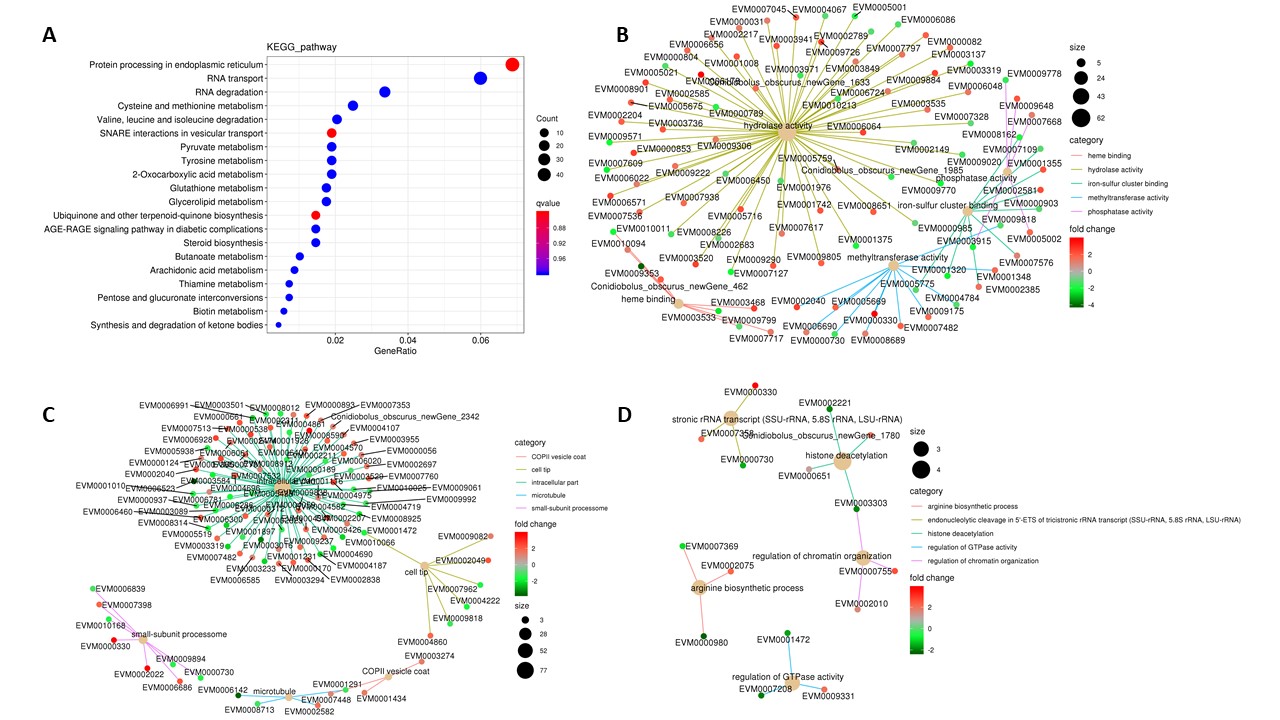


**Fig. S4** Overview of the GO and KEGG functional annotation of the protein-coding genes potentially regulated by 81 differentially expressed lncRNAs. (A) KEGG enrichment analysis of potential lncRNA-regulated protein-coding genes. (B) Interactive network of GO functional enrichment in the subcategory of molecular function. (C) Interactive network of GO functional enrichment in the subcategory of cellular component. (D) Interactive network of GO functional enrichment in the subcategory of biological process. (B-D) Red: upregulated (fold change ≥2), Green: downregulated (fold change ≤-2). Size of dots related to the gene number.

**Protein sequences of all the 30 small heat shock proteins and 2 upregulated HSP70s**

>EVM0009126 MSTRG5307.7 MSTRG5307.5

MFATLNNSPFVYYPKEFHPFVFTPEHYTPFNHHEKNTTPTTPQENNPQKQTINTNNVTINTTKPKEEKWPYVDITEFPNYYTIEAELPGFKKEDINISFDNVDNTLTLKGEYVYRPKKHEHIVIVEEADETNEDDNASISTSSVTDDGKHLLTERRSKKSFKRSFKVKHDILAEEIQADLTDGVLNLIIPKRVKKEDKKIRISIQ

>EVM0004881 MSTRG9187.1

MSLRRSNQENSLFHPFSSDHGLFSPLLHGSSYFQDLSPKVDVYSKEDKVTVNAELPGFKKEDLHIDFSDNLRQITISGTKKRDETHKEGDATYSERSYGSFSRTLSLPHGTR

>EVM0000913

MLGISISINEISSLQPFEHLHLSPLQHSFRNRGPLLTFNDNWDEFLNPKVEVIRKDQQTIVQAELAGFNKEDIQINVSEDQRQLVLSGESKRDKTHNEGEISYSEREYGSFTRSFTLPPGSKTEDIKAKFDNGLLEVIIPKEKLRNSNVKLLLIK

>EVM0008022

MSLSPLLTFNDNWDEFLNPKVEVIRKDQQTIVQAELAGFNKEDIQINVSEDQRQLVLSGESKRDKTHNEGEISYSEREYGSFTRSFTLPPGSKTEDIKAKFDNGLLEVIIPKEKLRNSNVKLLLIK

>EVM0003551

MSLRRSNQENSLFHPFSSDHGLFNLSPKVDVYSKEDKVTVNAELPGFKKEDLHIDFSDNLRQITISGTKKRDETHKEGDATYSERSYGSFSRTLSLPHGTRADDVKAKYDNGVLEIIIPRDTSSTQRKIQIS

>EVM0009984 MSTRG11196.3

MSLSRFLRSSLQPFEHLHLSPLQHSFRNGGPLLKFNNNWEEFLNPRVEVIKKDQQTIVQVELAGFNKEDIQINVSEDQRQLVLTGESKRDKTHNEGEISYSEREYGSFTRSFTLPSGSKTEDIKAKFDNGLLQVIIPKEETKEQQRQITID

>EVM0000402

MSLRRSNQENSLFHPFSNLSPKVDVYSKEDKVTVNAELPGFKKEDLHIDFSDNLRQITISGTKKRDETHKEGDATYSERSYGSFSRTLSLPHGTRADDVKASMIMVS

>EVM0008944 MSTRG5307.7 MSTRG5269.3 MSTRG5294.1 MSTRG5307.5

MFTTLNNNPFAHYPKELHPFVFTPDHYIPFNHKKNTTSTTQENNTEKQFTNDNNKIPLTDAAKSQEDKYPYVDMTEYSNYYTIEAELPGFRKEDVEISFDNHENIVIVEEADEANEDDNASVSTSSVTEDGKHLLVERRSKNFKRSFKVKQDILAEEIQADLTDGVLNLIIPKRFKKKIRK

>EVM0006922

MSLSRYLGSSLPFDSRIGYPHHHLLNYDPFFSDTSDYSFSPKVEVIRKEDETVVQAELAGFNKNDIHVNVSDDCRRLVIYGESKRDKSYQEGTVSYSERSYGSFTRSFNIPIDSKTDDIKASFNNGLLYVKVPRDVNKEHQQHTIAIE

>EVM0000723 MSTRG12801.2 MSTRG12808.1

MFTTLNNNPFTYYPKELHPFVFTQDHYTPFNCHKNDTMPTTPQVNNTEKQSVNANNKTPLANATKSQEDGWPYVDITEYPNCYTIEAELPGFKKEDIDISFDNIDNIITLKGEYIYRPKKHENIVIVEEADEANEDDNASVSTSSVTEDGKHLLVERRSKKNFKRSFKVKQDILAEEIQADLTDGVLNLIIPKRFKKKDKKIRISIQ

>EVM0006644

MYLTLDDLTPFLFYDESKNSKPLKRCQNKRTKRPERVQKRKLLHSNCPSININKTKNNSIKPDSSNPELQPTKTESCLWPKADIIEYSSYYQMELELPGFKKQDISIDYDNEENQLIIKGTYVYRPKKSEHIVTVEDDLDEDSSSDLTSISGASTSSEGTELLMERKSKKGFNRSFKIEKEIIEKEIKAELMDGVLSLILPKSTMKKNKKIRIDIQ

>EVM0003072

MSTQYTITDLFDQYSPVELLMLSHLNPEHKKGNKGAHKVQNRSKSIQVNLVENENEFQIQALIPGYEKSDLSIDYSSTNNSIIIETKVKEAADSEKEENKPKFLVKEFEPSKLHREVKLSSPVNPENISATLELGVLKLIVPKQNVEKVHRISIM

>EVM0009023

MELKVYTTKPACTSAFDNQSYIRYYFQNSECKPALKQDKWPKVDIVEYPNYYIIEAEVPGFKKENLDISFDNVENTITLKGEYVEKPNKHETAVLVENENENENNPEKENDNPESINSSSNAEYGKQLITERNSKKNFRRSFKVKQQIVIEEIEAELNDGILSLLIPKHIKEEDKETKIPIQ

>EVM0007432

MSLSRFLRSSLQPFEHLHLSPLQHSFRNRGPLLTFNDNWDEFLNPKVEVIRKDQQTIVQAELAGFNKEDIQINVSEDQRQLVLSGESKRDKTHNEGEISYSEREYGSFTRSFTLPPGSKTEDIKAKFDNGLLEVIIPKGEIKEQQRQITID

>EVM0008914

MSNPNFFNQFSTDIHGKVKNMIQQNMNSFSDAQQNFNSFSSFNTGFQPSFETEDKDNEVLVTLKVSGFTKDDLKVSLLANHLSVSGNSNTNSQSASSFGNAFSSSSNAFSRSMELPNKYVYDGFTYEMNDSVLKVVVKKKN

>EVM0006259

MSLSRFLRSSLQPFEHLHLSPLQHSFRNRVLNPKVEVIRKDQQTIVQAELAGFNKEDIQINVSEDQRQLVLSGESKRDKTHNEGEISYSEREYGSFTRSFTLPPGSKTEDIKAKFDNGLLEVIIPKGEIKEQQRQITID

>EVM0004909 MSTRG11196.3

MSLSRFLRSSLQPFEHLHLSPLQHSFRNRGPLLTFNDNWDEFLNPKVEVIRKDQQTIVQAELAGFNKEDIQINVSEDQRQLVLSGESKRDKTHNEGEISYSEREYGSFTRSFTLPPGSKTEDIKAKFDNGLLEVIIPKGEIKEQQRQITID

>EVM0004897

MSLSRFLKNRGPLLTFNDNWDEFLNPKVEVIRKDQQTIVQAELAGFNKEDIQINVSEDQRQLVLSGESKRDKTHNEGEISYSEREYGSFTRSFTLPPGSKTEDIKAKFDNGLLEVIIPKGEIKEQQRQITID

>EVM0002060

MATKYPHQWAESANSYYFQVKLPGFENDDIDINIDHGTLKISADKAKEEEDEEEINNRKSNINEKEAFKYNVDLPADKICLSKVHAFIQQGLLIVKCPKLTKSDLPSLKVQLLKNNL

>EVM0000226

MELKVYTTKPACTSAFDNQSYIRYYFQNSECKPALKQDKWPKVDIVEYPNYYIIEAEVPGFKKENLDISFDNVENTITLKGEYVEKPNKHETAVLVENENENENNPEKENDNPESINSSSNAEYGKQLITERNSKKNFRRSFKVKQQIVIEEIEAELNDGILSLLIPKHIKEEDKETKIPIQ

>EVM0001228

MFKVYATKPACISAFDDQSYVRYYFQNSECKPALKQDKWPNVDIVEYPNYYIIEAEVPGFKKENLDVSFDNVENTITLKGEYVEKPNKHETTVLVENENENNPQKENENPESINTSSNAKYGKQLITERNSRKDFKRSFKVKQQIVIEEIEAGLNDGVLSLLIPKHIKEEDKETKIPIQ

>EVM0006614

MSLRRSNQENSLFHPFSNLSPKVDVYSKEDKVTVNAELPGFKKEDLHIDFSDNLRQITISGTKKRDETHKEGDATYSERSYGSFSRTLSLPHGTRADDVKAKYDNGVLEIIIPRDTSSTQRKIQIS

>EVM0004943 MSTRG9187.1

MSLRRSNQENSLFHPFSSDHGLFSPLLHGSSYFQDLSPKVDVYSKEDKVTVNAELPGFKKEDLHIDFSDNLRQITISGTKKRDETHKEGDATYSERSYGSFSRTLSLPHGTRADDVKAKYDNGVLEIIIPRDTSSTQRKIQIS

>EVM0004933

MFTTLNNNPFTYYPKELHPFVFTQDHYTPFNCHKNDTMPTTPQVNNTEKQSVNANNKTPLANATKSQEDGWPYVDITEYPNCYTIEAELPGFKKEDIDISFDNIDNIITLKGEYIYRPKKHENIDGKHLLVERRSKKNFKRSFKVKQDILAEEIQADLTDGVLNLIIPKRFKKKDKKIRISIQ

>EVM0000546

MSNPNFFNQFSTDIHGKVKNMIQQNMNSFSDAQQNFDSFSSFNTGFQPNFETEDKDNELLVTLKVSGFTKDDLKVSLLANHLSVSGNSNMNSQSASSFGNAFSSSSNAFSRSMELPNKYIYDGFTYEMNDNVLKIVVKKKN

>EVM0007322 MSTRG11196.3

MSLRRSNQENSLFHSFGGDHGLFSPLLHAGSNYFQDLSPRVDVYSKEDKVTVHAELPGFKKEDLHVDFSDNLRQITISGTKKRDETHKEGDATYSERSYGNFSRTLSLPHGTRADDVKAKYDNGVLEIIIPRDTSSTQHNIINTINFIDEDRENDKEKERQREDEKEEEREDYDELDEFENKNKNKNGKEDHISECQQLHQVLVQLSKMYGYAKFCEIKASITSSKFDDIVLPSILVYRNNQLEHNLIRFIDEFNSKDINLQNTEAVLLRLGVLSSSDKFLIEE

>EVM0001986 MSTRG12801.2 MSTRG12808.1

MFATLNNNPFVYYPKEFHPFVFTPEHYTPFNHHEKNTTPTTPQENNPQKQTINTNNNVTINTTKPKEEKWPYVDITEFPNYYTIEAELPGFKKEDINISFDNVDNTLTLKGEYVYRPKKHEHIVIVEEADETNEDDNASISTSSVTDDGKHLLTERRSKKSFKRSFKVKHDILAEEIQADLTDGVLNLIIPKRVKKEDKKIRISIQ

>EVM0008368 MSTRG12801.2 MSTRG12808.1

MFATLNNSPFVYYPKEFHPFVFTPEHYTPFNHHEKNTTPTTPQENNPQKQTINTNNNVTINTTKPKEEKWPYVDITEFPNYYTIEAELPGFKKEDINISFDNVDNTLTLKGEYVYRPKKHEHIVIVEEADETNEDDNASISTSSVTDDGKHLLTERRSKKSFKRSFKVKHDILAEEIQADLTDGVLNLIIPKRVKKEDKKIRISIQ

>EVM0007115 MSTRG12801.2 MSTRG12808.1

MFTTLNNNPFAHYPKEFHPFIFTPDHYIPFNHKKNTTSTTQENNTEKQLTNDNNKIPLTNAAKSQEDKYPYVDMTEYSNYYTIEAELPGFKKEDIEISFDNVDNIITLKGEYTYRPKKHENIVIMEEADETNEDDNVSVSTSSVNDDGKHLLIERKLKKSFKRSFKVKHDILAEEIQADLTDGVLNLIIPKRFKKENKKIRIPIQ

>EVM0004757 MSTRG9187.1 MSTRG5294.1

MSLSRFLRSSLQPFEHLHLSPLQHSFRNRGPLLTFNDNWDEFLNPKVEVIRKDQQTIVQAELAGFNKEDIQINVSEDQRQLVLSGESKRDKTHNEGEISYSEREYGSFTRSFTLPPGSKTEDIKAKFDNGLLEVIIPKGEIKEQQRQITID

>EVM0008702 MSTRG12823.2

MSVVGIDLGNLSSYIAVARNRGIDVITNEVSNRDTPSLVSFGTKQRYIGESAKTQEVSNFKNTVGSLKRIIGRSFNDPEIEVEKRFINAELVDVDGQVGVKVNFKGEPTTFTATQLMGMYLGKLRDTAANELKLPCSDVVVSVPGWFNDRQRRALLDACQIANLNCLRIMNEITASALGYGITKTDLPETEPRNVIIVDVGHSSYSVAAVSYIKGQLNVKATAYDNKVGGRYIDELLVNHFAQVFKEKYKIDILSNPKATFRLRTGVEKLKKVLSANSQAPLNIESIMEDRDVSALMKREEFEELIQDQLERLAKPLSDVIAEVGWGTDQIYSVEIVGGTSRIPTVKEKISSLLGKELSFTLNQDEAVSRGCALQCAILSPVFKVRDFSVTDILNFPVKFTWTSTEGGADSEVDVFSFKNPVPSSKILTFYRKEPFEFQANYSNPKKLPAGTNPWIGTFSVKNVTSLKDGELSTVKVKARVNLHGVINVESAHIAEEIIQTEEPAEGDNPEDPPKTKKVIKKHDLPVVSQTNSLDENILGRYKEVENDMYASDKLVTDTEHAKNALEEYVYDTRSKLSGAYSSYIDPTIKDSFIASLNEVEDWLYGDGEDTTKSVYVSKIEELNQIGAPVIARYRESSQRPFAAENLKKEIERYRNLAIGGDVKYDHIAPEELEKIVNKCNELEKWLKTELATIESLPKYETPKVTSAQIKQSQDQLQYLANPILSKPKPAPKVEEPKPASGEQSPEKATEEPKQEQDDMDID

>EVM0000047 MSTRG1788.17

MSKSRAIGIDLGTTYSCVGVWQNDRCEIIANDQGNRTTPSYVAFTDSERLIGDAAKNQVAMNPYNTVFDAKRLIGRRFDEPEVQSDMKHWPFKVIDKSTKPIIQVEYKGETKQFTPEEISSMVLIKMRETAEAYLGTTVTDAVVTVPAYFNDSQRQATKDAGAIAGLNVLRIINEPTAAAIAYGLDSKTTGEKNVLIFDLGGGTFDVSLLSIEDGIFEVKATAGDTHLGGEDFDNRLVNHFMQEFKRKNKKDISSNARAMRRLRTACERAKRTLSSAAQTSIEIDSLFEGIDFYTSITRARFEELCQDLFRSTVDPVEKVLRDSKIDKSAVNEIVLVGGSTRIPKIQKLVSDYFNGKEPNKSINPDEAVAYGAAVQAAILTGDTSEKTQDLLLLDVAPLSLGIETAGGVMTPLIKRNTTVPTKKSETFSTYADNQPGVLIQVFEGERARTKDNNLLGKFELSGIPPAPRGVPQIEVSFDIDANGILNVTAADKSTGRSNKITITNDKGRLSKEDIERMVAEAEKYKSEDEAAAARISAKNGLESYAYNLRNTLNDEKVAGKLEAADKEKLEAAIKEATEWLDNSHEASKEEYEERQKELEGVANPIMAKIYAAGGAPGAEGGFPGGFPGGAPGGAPGGFPGAPPAGPGADGPTIEEVD
